# Supplementary material for: Pyrazinacenes exhibit on-surface oxidation-state-dependent conformational and self-assembly behaviours
Source: Commun Chem. 2021 Mar 10;4:29. doi: 10.1038/s42004-021-00470-w (PMC9814942; doi:10.1038/s42004-021-00470-w)
Supplement: Supplementary file 1 — Supplementary Information [file 42004_2021_470_MOESM1_ESM.pdf]

Supplementary Information

Pyrazinacenes exhibit on-surface oxidation-state-dependent conformational and self-assembly behaviours

David Miklík, S. Fatemeh Mousavi, Zuzana Hloušková, Anna Middleton, Yoshitaka Matsushita, Jan Labuta, Aisha Ahsan, Luiza Buimaga-Iarinca, Paul A. Karr, Filip Bureš, Gary J. Richards, Pavel Švec, Toshiyuki Mori, Katsuhiko Ariga, Yutaka Wakayama, Cristian Morari, Francis D'Souza, Thomas A. Jung and Jonathan P. Hill

Supplementary Methods.

Synthesis of Precursors

2,3-Dichloro-6,7-diphenyl-1,4,5,8-tetraazanaphthalene (X = Cl, **3**). Benzil (210 mg, 1 mmol) and 2,3-diamino-5,6-dichloropyrazine (190 mg, 1.05 mmol) were dissolved in a mixture of dry 1,4-dioxane (10 mL) and glacial acetic acid (2 mL). The solution was heated under reflux for 12 h. The reaction mixture was allowed to cool then poured into 150 mL of water followed by partitioning of the resulting mixture with DCM (2 × 40 mL). The combined extracts were washed with 30 mL of brine, dried over anhydrous Na_2SO_4 then the solvents were evaporated under reduced pressure. The residue was purified by column chromatography (silica, DCM) to give the product as a yellow crystalline solid. Yield: 307 mg (87 %). ^{1}H NMR (300 MHz, $CDCl_3$, 25 °C) δ = 7.34 – 7.4 (m, 4 H, Ar*H*), 7.43 – 7.48 (m, 2H, Ar*H*), 7.63 – 7.67 (m, 4 H, Ar*H*) ppm. ^{13}C NMR (75 MHz, $CDCl_3$, 25 °C) δ = 128.5, 130.2, 130.4, 137.0, 142.2, 149.2, 158.2 ppm. FTIR (ATR) $\tilde{\nu}$= 3109, 3075, 2918, 2849, 2558, 1557, 1451, 1401, 1338, 1244, 1234, 1163, 1082, 1068, 1007, 968 cm^{-1}. HR-MALDI-MS (DHB): m/z calc. for $C_{18}H_{10}Cl_2N_4$ $[M]^+$: 352.0283; found 352.0283.

2,3-Diamino-6,7-diphenyl-1,4,5,8-tetraazanaphthalene, **7**. Dry ammonia gas was bubbled through a solution of 2,3-dichloro-6,7-diphenyl-1,4,5,8-tetraazanaphthalene (200 mg, 0.57 mmol) in DMF (7 mL) for 15 min at RT. The reaction mixture was then heated at 90°C in a sealed tube for 12 h. Solvents and excess ammonia were then removed under reduced pressure. The solid residue was ultrasonicated with water (20 mL), the solid was filtered, washed with ethyl-acetate (20 mL) then hexane (20 mL) giving the product as a yellow crystalline solid. Yield: 100 mg (56 %). ^{1}H NMR (300 MHz, DMSO-d_6, 25 °C) δ = 7.29 – 7.31 (m, 6H, Ar*H*), 7.35 – 7.39 (m, 4H, Ar*H*), 7.45 (br s, 4H, N*H₂*) ppm. ^{13}C NMR (75 MHz, DMSO-d_6, 25 °C) δ = 127.7, 127.8, 129.6, 139.5, 141.5, 146.8, 148.3 ppm. FTIR (ATR) $\tilde{\nu}$ = 3452, 3314, 3222, 3138, 2028, 1628, 1470, 1381, 1212, 1070, 1025 cm^{-1}. HR-MALDI-MS (DHB): m/z $[M + 2H]^+$ calcd for $C_{18}H_{14}N_6$: 316.1431; found 316.1429.

2,3-Dichloro-6,7-di(4-tert-butylphenyl)-1,4,5,8-tetraazanaphthalene. 4,4´-Di-tert-butylbenzil (323 mg, 1 mmol) and 2,3-diamino-5,6-dichloropyrazine (190 mg, 1.05 mmol) were dissolved in a mixture of dry 1,4-dioxane (10 mL) and glacial acetic acid (2 mL). The solution was heated under reflux for 12 h. The reaction mixture was then allowed to cool, poured into water (150 mL) and extracted with DCM (40 mL). The extract solution was washed with of brine (30 mL), dried over ahydrous Na_2SO_4, then solvents were evaporated under reduced pressure and the residue was purified by column chromatography (silica; DCM, hexane 5/1) to give the product as a yellow crystalline solid. Yield: 255 mg (55 %). ^{1}H NMR (300 MHz, $CDCl_3$, 25 °C) δ = 1.31 (s, 18H, C*H$_3$*), 7.38 (d, ^3J = 8.4 Hz, 4H, Ar*H*), 7.63 (d, ^3J = 8.4 Hz, 4H, Ar*H*) ppm. ^{13}C NMR (75 MHz, $CDCl_3$, 25 °C) δ = 31.2, 34.9, 125.4, 130.0, 134.3, 142.1, 148.7, 154.1, 158.1 ppm. FTIR (ATR) $\tilde{\nu}$ = 2962, 2926, 2868, 1729, 1604, 1461, 1333, 1235, 1166, 1112, 1008 cm^{-1}. HR-MALDI-MS (DHB): m/z $[M]^+$ calcd for $C_{26}H_{26}Cl_2N_4$: 466.1691; found 466.1692.

2,3-Diamino-6,7-di(4-tert-butylphenyl)-1,4,5,8-tetraazanaphthalene. Dry ammonia gas was bubbled through a solution of 2,3-dichloro-6,7-di(4-tert-butylphenyl)-1,4,5,8-tetraazanaphthalene (300 mg, 0.65 mmol) in DMF (10 mL) for 15 min at RT. The reaction mixture was heated at 90°C in a sealed tube for 12 h. Solvents and excess ammonia solution was removed using reduced pressure. The product was isolated using column chromatography (silica; $CHCl_3$, 8% MeOH) to give product like a yellow crystalline solid. Yield: 125 mg (45 %). ^{1}H NMR (300 MHz, DMSO-*d$_6$*, 25 °C) δ = 1.33 (s, 18H, C*H$_3$*), 7.34 – 7.43 (m, 12H, Ar*H* + N*H$_2$*) ppm. ^{13}C NMR (75 MHz, $CDCl_3$, 25 °C) δ = 31.0, 34.1, 124.42, 129.1, 136.7, 141.2, 146.6, 148.1, 150.1 ppm. FTIR (ATR) $\tilde{\nu}$ = 3435, 3316, 3235, 2959, 2864, 1700, 1520, 1435, 1365, 1333, 1268, 1207, 1117, 1016 cm^{-1}. HR-MALDI-MS (DHB): m/z $[M + 2H]^+$ calcd for $C_{26}H_{30}N_6$: 428.2688; found 428.2677.

Supplementary Figures.

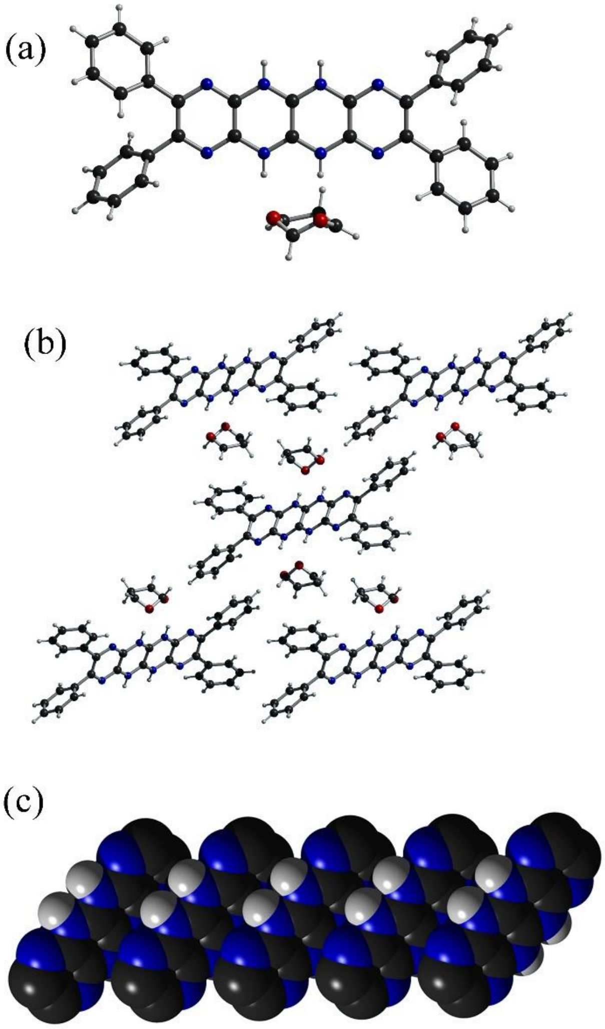

Supplementary Figure 1. X-ray crystal structure of **2**. (a) Single molecule with disordered tetrahydrofuran hydrogen-bonded at disordered NH groups, (b) Viewed down a axis. (c) Space-filling model of a slipped stack of the chromophore moiety

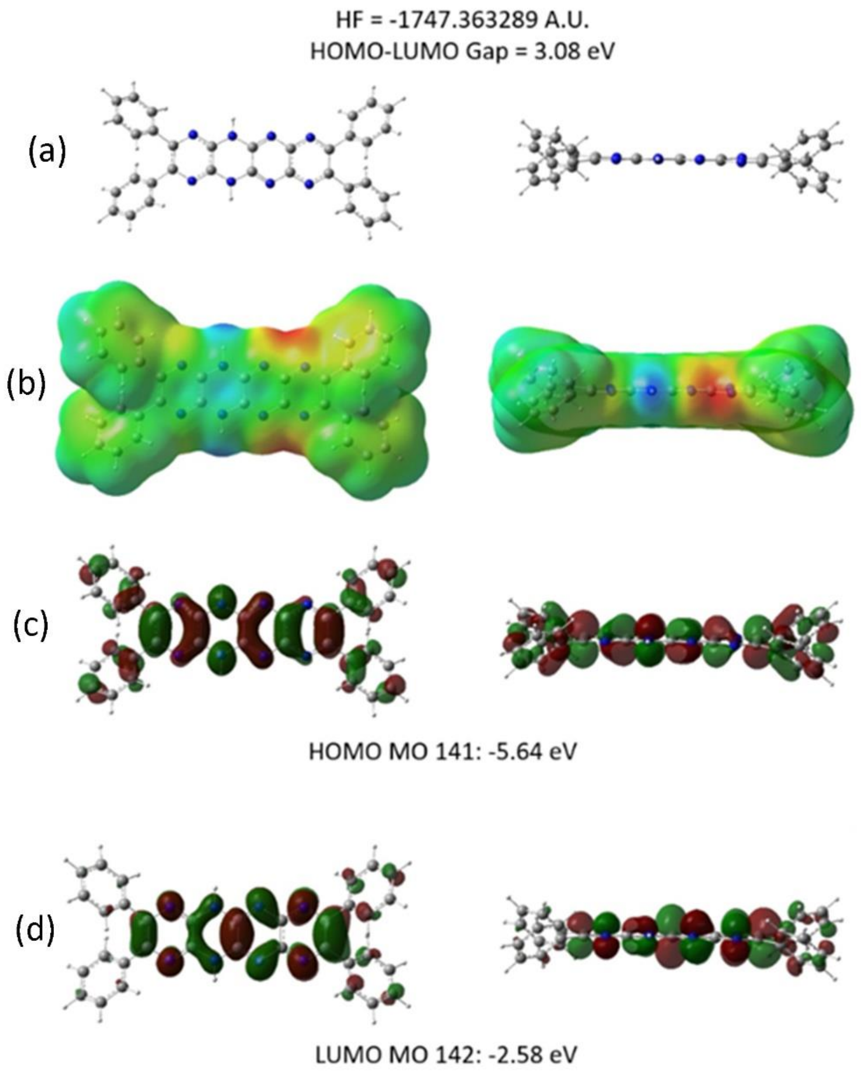

Supplementary Figure 2. DFT calculations for compound **2**. (a) Molecular structure. (b) Surface charge. (c) Highest occupied molecular orbital (HOMO), and (d) lowest unoccupied molecular orbital (LUMO).

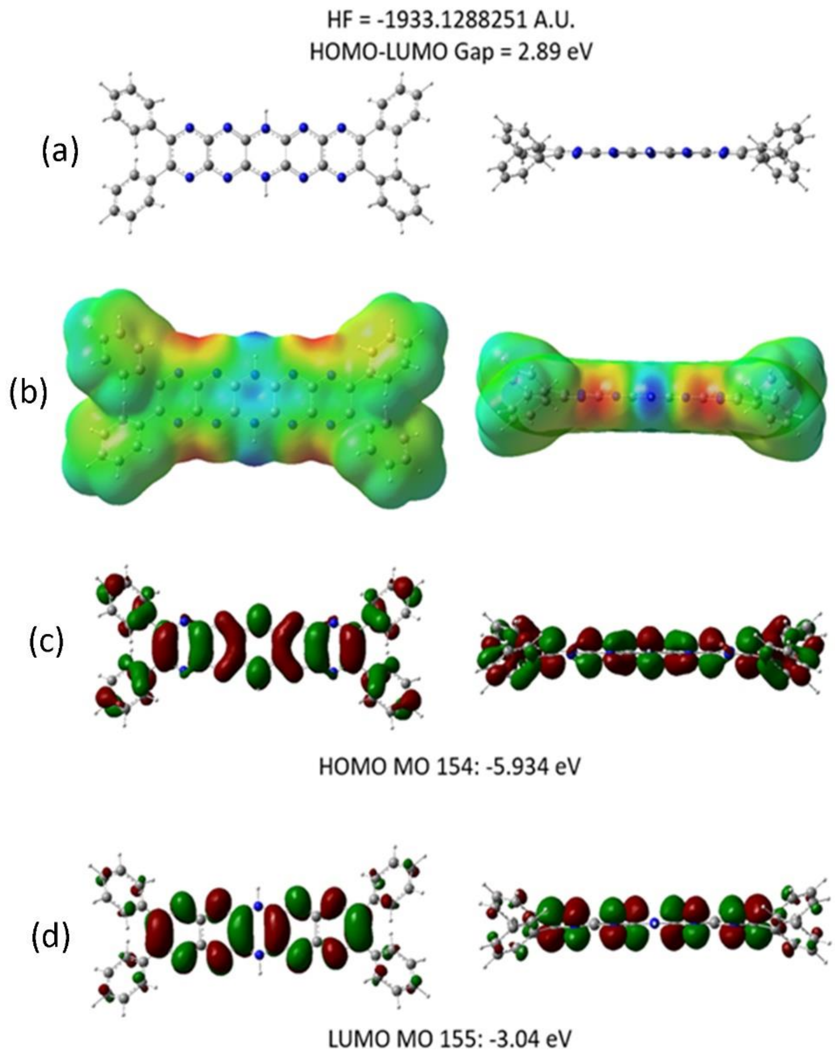

Supplementary Figure 3. DFT calculations for compound **1**. (a) Molecular structure. (b) Surface charge. (c) Highest occupied molecular orbital (HOMO), and (d) lowest unoccupied molecular orbital (LUMO).

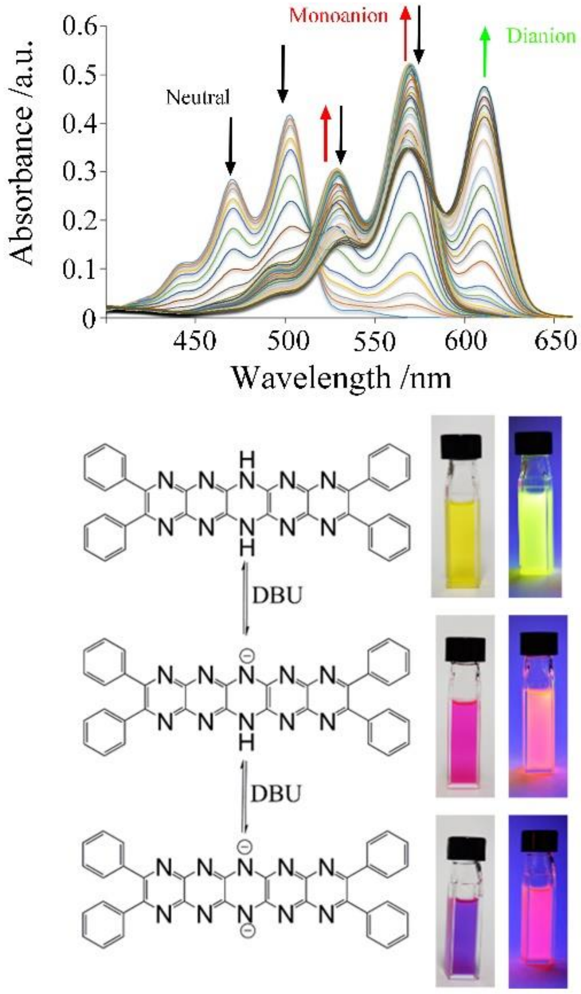

Supplementary Figure 4. UV/Vis titration of **1** with 1,8-diazabicyclo[5.4.0]undec-7-ene (DBU). Spectral changes can be fit according to a double deprotonation process with easy first deprotonation ($K_1 > 3 \times 10^7$ M^{-1}) and more difficult second process ($K_2 = 1.7$ (± 0.1) $\times 10^6$ M^{-1}). Proposed chemical structures and photographs of the solutions obtained during titration are also given for samples under daylight and under UV light (λ_{ex} = 365 nm).

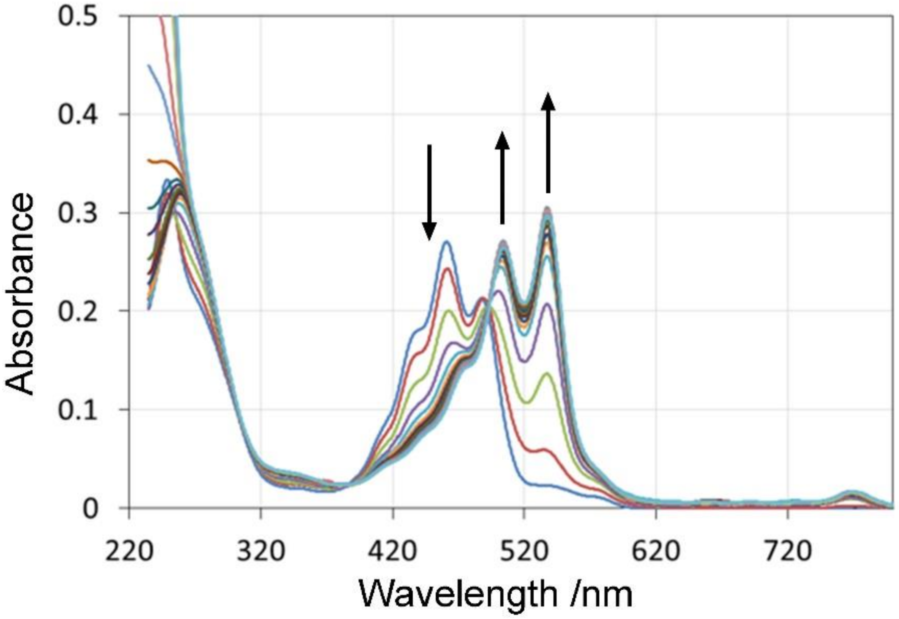

Supplementary Figure 5. Titration of **2** with DBU. Spectral changes can be fit according to a single deprotonation process ($K = 6.5 \times 10^5$ M^{-1}). In this case, the dianion could not be observed due to the lower acidity of the amine protons in **2**.

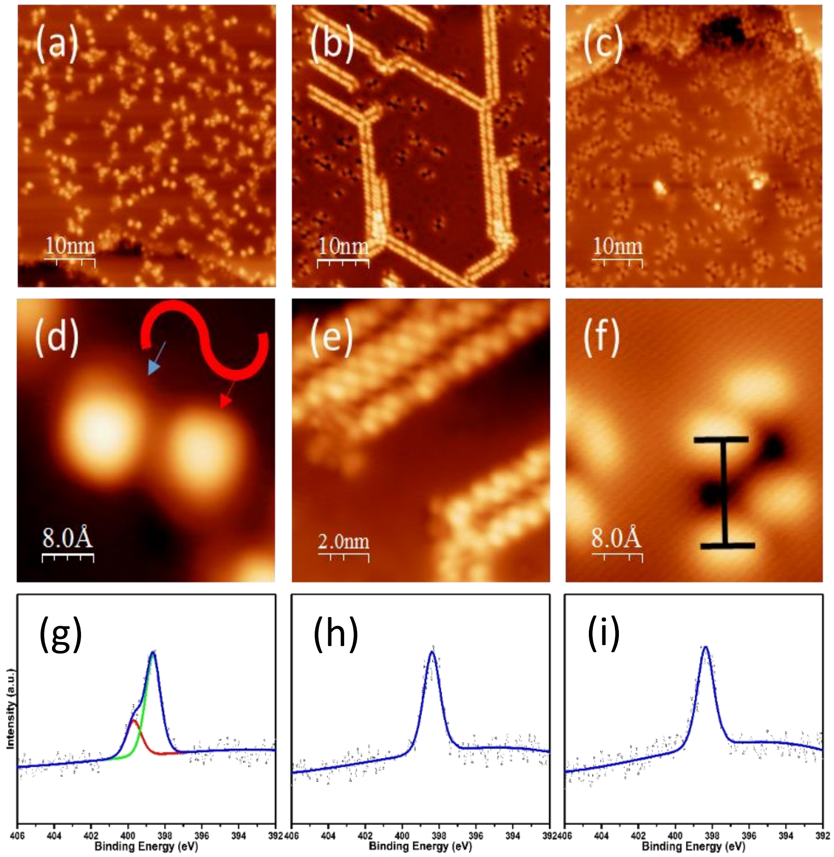

Supplementary Figure 6. Scanning tunneling micrographs of **1** on Cu(111). (a) **1** distributed as found, predominantly with a double-lobe form. In contrast to **2**, other forms can also be found at low concentration and are largely eliminated following subsequent annealing. (b) Line structures formed upon annealing of **1** at 150 °C on Cu(111). (c) Double-lobed two-dark-spot structures dominate after annealing at 300 °C. (d-f) Magnified images of (a-c), respectively. (d) Chiral double-spot profile of **1** similar to that found for **2** (Fig. 2d). (e) Line structures with phenyls and acene backbones clearly recognizable. (f) Double-lobed/two-dark-spot structure obtained for **1** due to dehydrogenation/cyclodehydrogenation (see main text). (g-i) N1s XP spectra of **1** on Cu(111): (g) as-deposited as in (a),(d); (h) after annealing at 150 °C as in (b),(c); (i) after annealing at 300 °C as in (c),(f). STM data information: (a) 50 nm × 50 nm, 10 pA, 1 V. (b) 50 nm × 50 nm, 50 pA, 300 mV. (c) 50 nm × 50 nm, 10 pA, 1 V. (d) 4 nm × 4 nm, 10 pA, 1 V. (e) 10 nm × 10 nm, 50 pA, 300 mV. f) 4 nm × 4 nm, 50 pA, 300 mV.

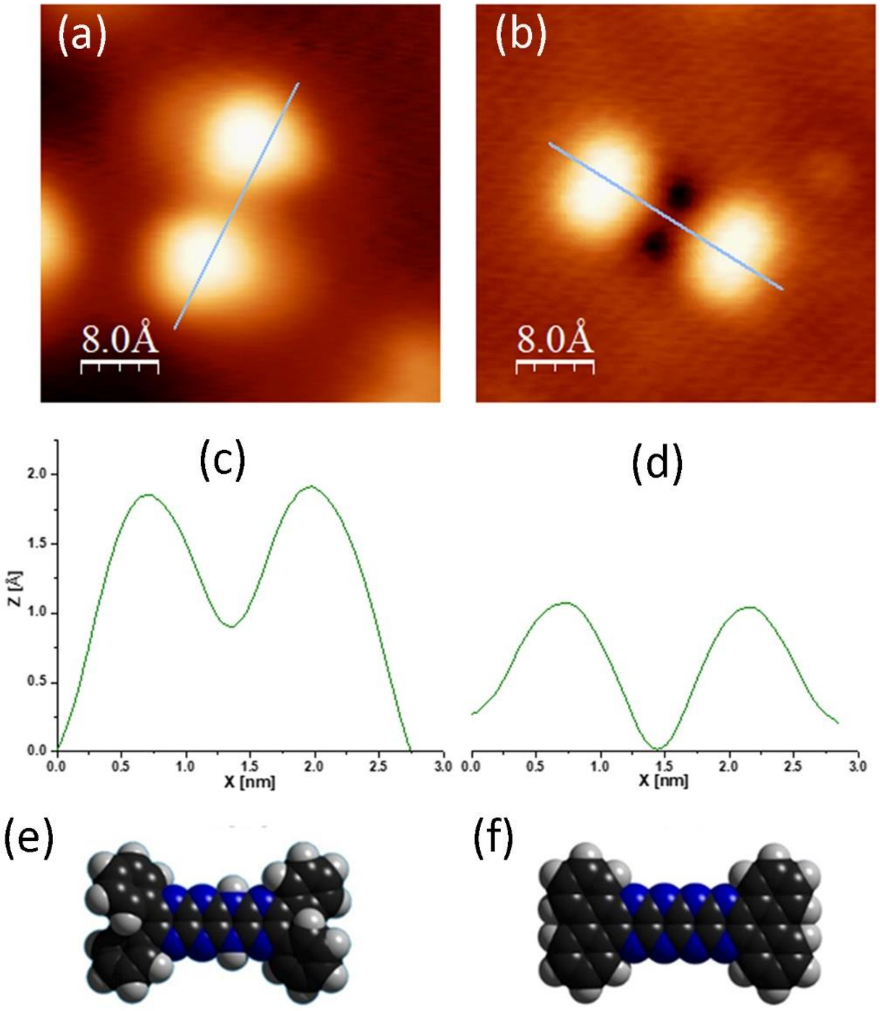

Supplementary Figure 7. Molecule profiles for **2** prior to and following annealing (300 °C) (a) As-deposited molecule. (b) after annealing at 300 °C. (c), (d) Corresponding distance profiles for lines given in (a) and (b), respectively. Space-filling models of (e) **2** and (f) the species present after annealing (**2**-ox$_2$). STM data information: a) 4 nm × 4 nm, 10 pA, 1 V. b) 4 nm × 4 nm, 10 pA, 1 V.

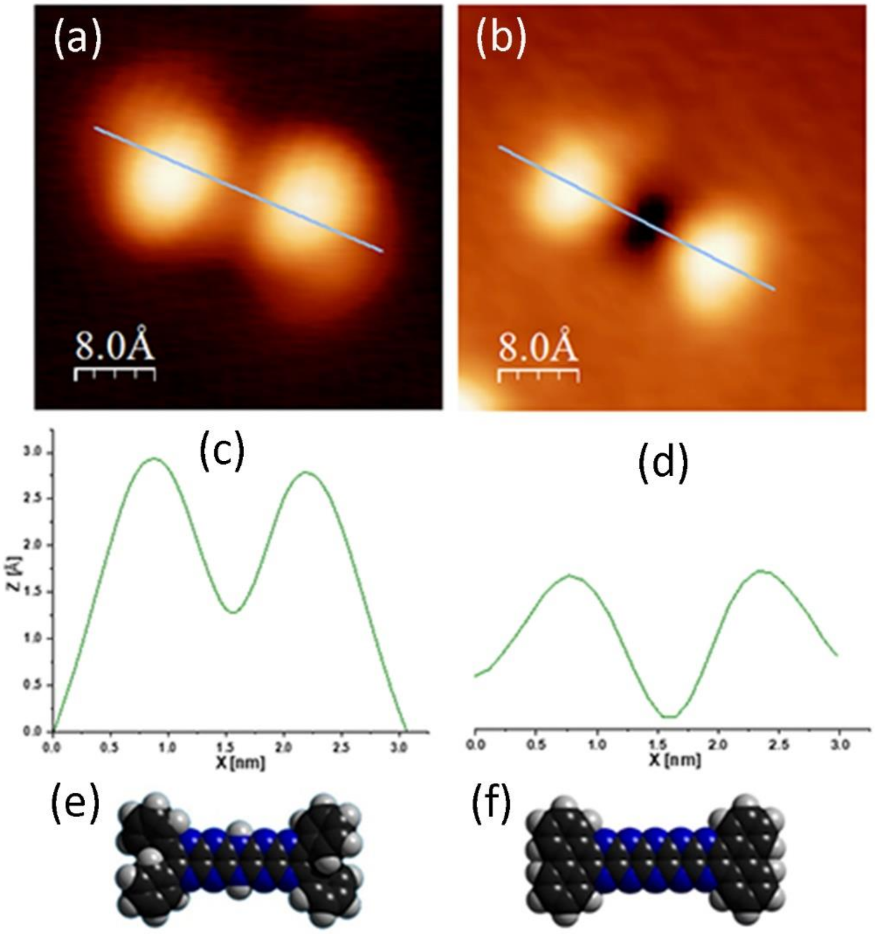

Supplementary Figure 8. Molecule profiles for **1** prior to and following annealing (300 °C). (a) As-deposited molecule. (b) after annealing at 300 °C. (c), (d) Corresponding distance profiles for lines given in (a) and (b), respectively. Space-filling models of (e) **1** and (f) the species present after annealing (**1**-ox$_2$). STM data information: a) 4 nm × 4 nm, 10 pA, 1 V. b) 4 nm × 4 nm, 10 pA, 1 V.

(a)

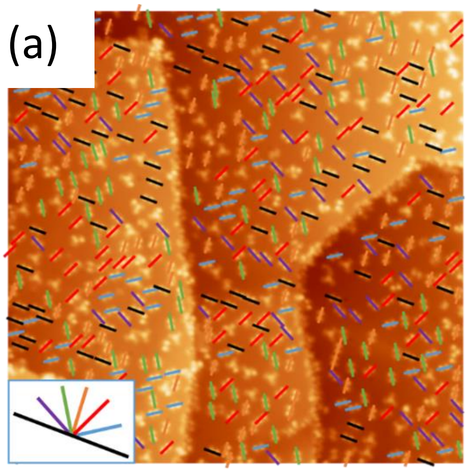

direction	Relative percentage
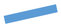	14.96%
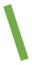	16.29%
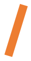	20.76%
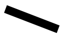	18.53%
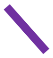	12.72%
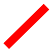	16.74%

(b)

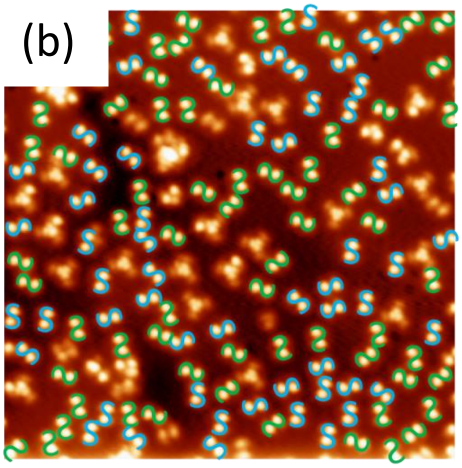

Supplementary Figure 9. Analysis of surface chirality for **2**. a) Molecules align along six directions on the threefold symmetric Cu(111) substrate. This implies that the 2 chiral conformers take a mirror orientation along the 3 principal directions of the substrate. Note that the determination of the angles is of limited precision. In the table on the right the number of molecules oriented in one or the other direction has been listed. b) Chirality identification. Molecules are adsorbed in two different chiral conformations and have been marked with blue and green 'S' and mirror 'S' features depicting the molecules of opposing chirality. Both enantiomers are present with about equal probability. Figure information: a) 100 nm × 100 nm; 10 pA, 1 V; b) 50 nm × 50 nm; 10 pA, 1 V.

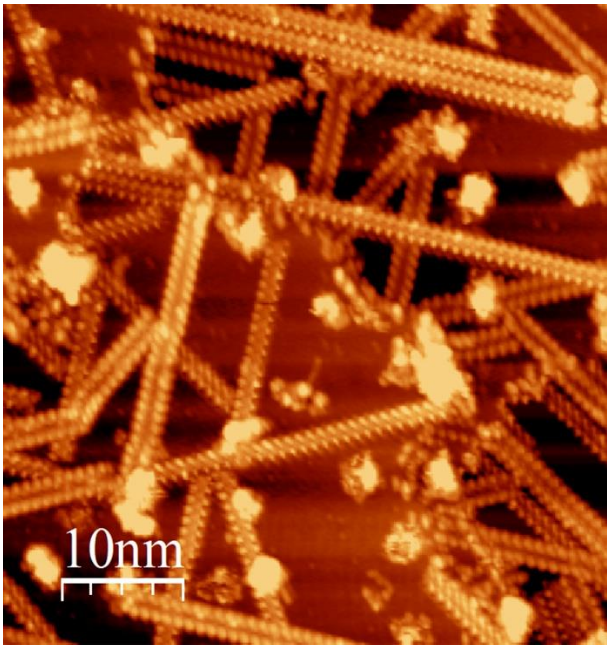

Supplementary Figure 10. STM images of **2** on Cu(111) after a period of 24 h at ambient temperature revealing that line formation occurs even at low temperatures.

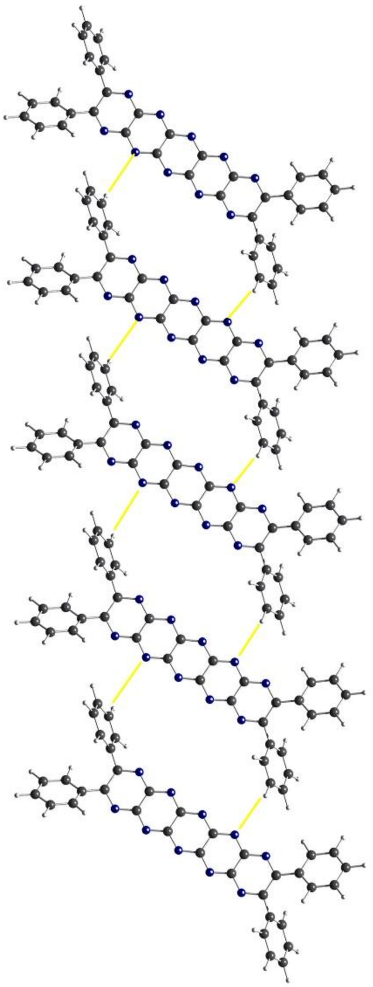

Supplementary Figure 11. Packing structure within lines of **1** after self-assembly on Cu(111). The major structure directing factor is C-H…N hydrogen bonding, which is a also a major structure-director in crystals of pyrazinacenes (see Supplementary Fig. S12 overleaf).

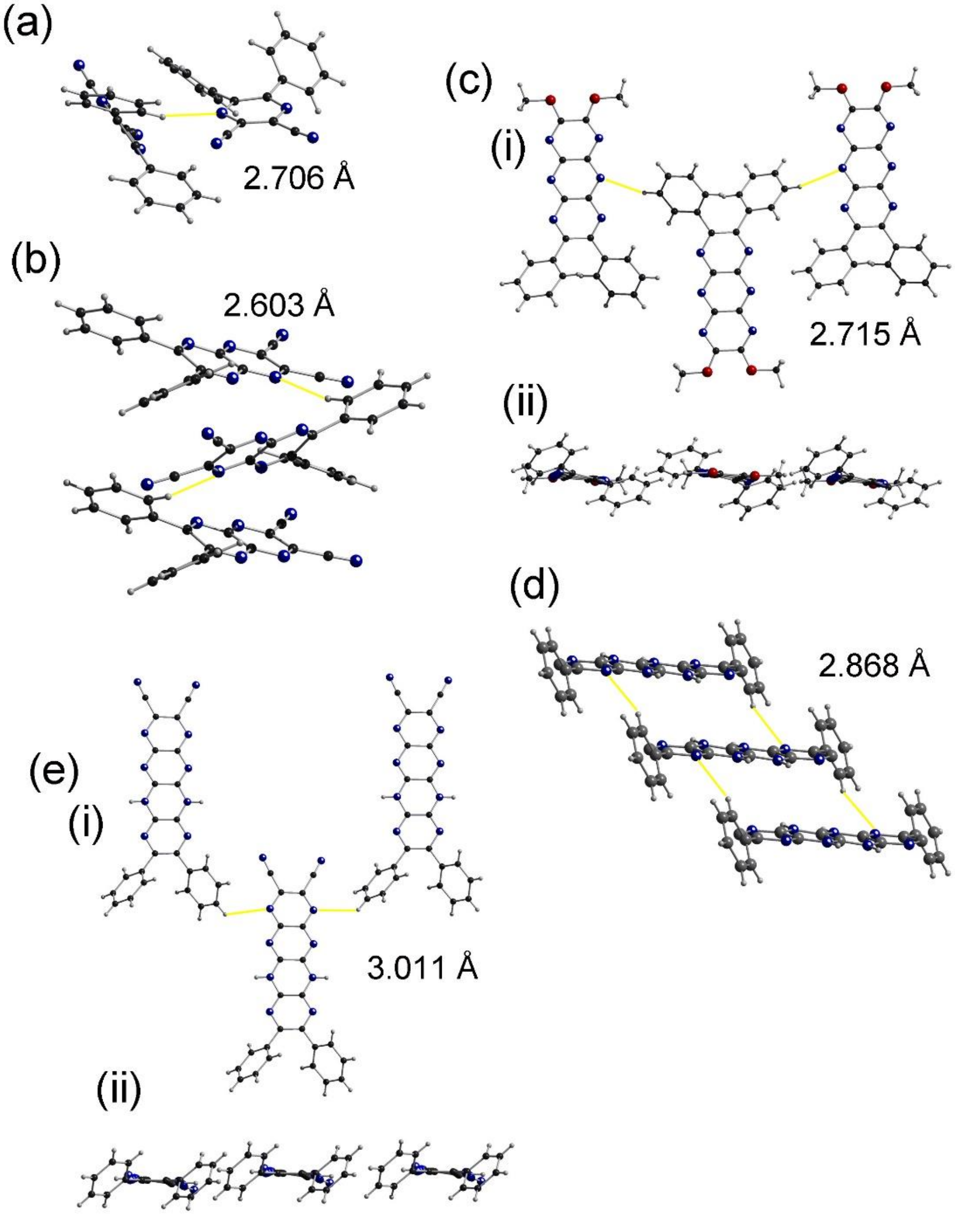

Supplementary Figure 12. C-H…N H-bonding distances in X-ray crystal structures of other pyrazinacenes. Distances quoted are for close approach of proton to N atoms. (a) 2,3-Diphenylpyrazine-5,6-dicarbonitrile, (b) 2,3-diphenyl-1,4,5,8-tetraazanaphthalene-6,7-dicarbonitrile, (c) (i) 2,3-diphenyl-1,4,5,8,9,10-hexaaza anthracene-6,7-dicarbonitrile. (ii) Side view revealing edge-wise packing. (d) **2** (this work) and (e) (i) 2,3-diphenyl-5,12-dihydro-1,4,5,6,7,10,11,12-octaazatetracene. (ii) Side view revealing edge-wise packing. All data taken from Reference 20 in the main manuscript.

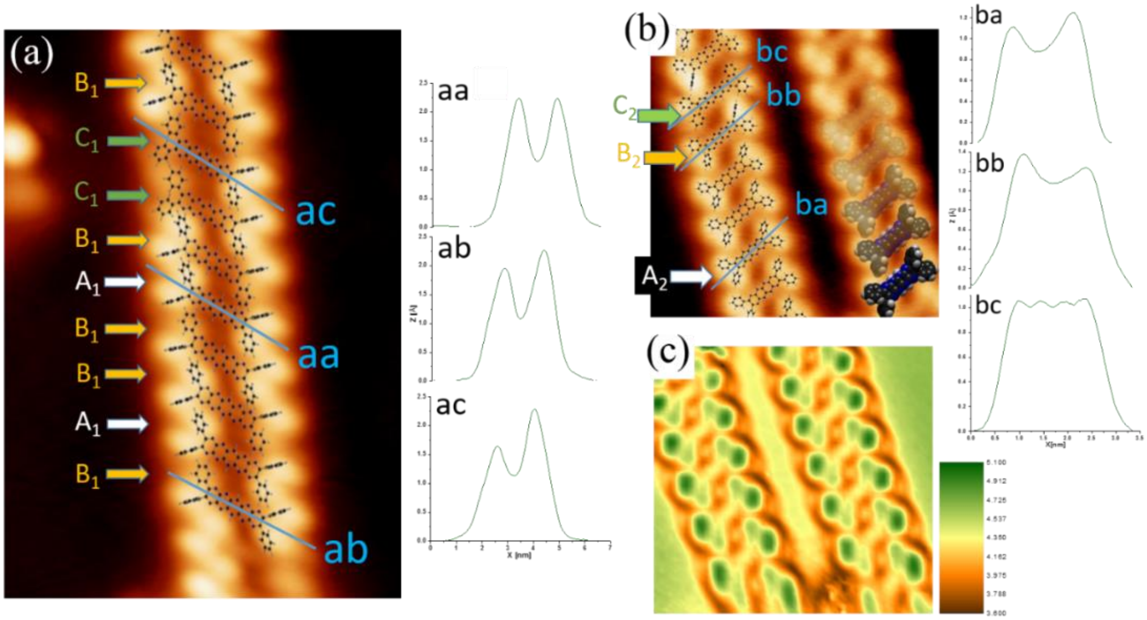

Supplementary Figure 13. STM profile data for self-assembled line structures of (a) **1** and (b) **2**. The line structures contain 'defects' which we identify as molecules taking different than the calculated minimum energy configuration. These conformations differ in the degree of in-plane/out-of-plane rotation of a subset of the 4 phenyl residues. A_1, B_1 and C_1 denote three different conformations of **1** arranged within a linear array; the corresponding height profiles shown at right reveal depressed points due to phenyl group rotation (lower dihedral angles between phenyls and pyrazinacene). Molecular profiles aa, ab and ac are described by the correspondingly labeled lines in the STM image (a). **2** similarly shows 3 conformations A_2, B_2 and C_2 with the height profiles also shown at the right of the STM image. STM images contain the respective packing models for **1** and **2**. (c) Work function map of **2**. The work function probes the local charge modification on the molecules by measuring current-distance I(z) plots at every point of the sample. From these it appears that the electronegative phenyls are imaged with higher electronegativity. Also the flexed rotation of the phenyl groups with respect to each other and the substrate is clearly shown. Due to the high electronegativity of the molecules, the surrounding borders of the chains are electron depleted and are identified by their lower electronegativity values. Molecular profiles aa, ab and ac are again described by the correspondingly labeled lines in the STM image (b).

Supplementary Note 1. Variation in the height profiles of molecules contained within the line structures can be assigned to conformational variation of the phenyl substituents of the compounds, i.e. different dihedral angles between the planes of phenyl groups and the plane of the pyrazinacene backbone. For **2**, variation in the phenyl group conformation is relatively rare and appears as 'defects' in the line structures. The observed defects within the arrays correspond to different conformers being included in the arrays; the STM profile height is lower due to rotation of the phenyl groups of one molecule. Possibly in order to reduce stresses within the array, the molecules adjacent to this defect rotate their phenyl groups and are of brighter STM contrast and greater height, as shown in Supplementary Fig. S13b (Conformations B_2 and C_2). Therefore, there exist three different conformations of **2** (indicated in the STM image overlay of Supplementary Fig. S13b) although that closely resembling the crystal structure conformation (Conformation A_2) dominates. In contrast, phenyl group dihedral angle variation in **1** (Figure Supplementary S13a) is significantly more common although there appears again to be three possible

conformations. The contrast of STM images of the line structure of **1** suggests that conformations with larger dihedral angles between phenyl groups and pyrazinacene backbone are preferred. It is difficult to specify these angles (suggested values are given in Supplementary Figure S13) but again rotation of the phenyl rings leads to their different heights in STM and allowed us to identify three distinct conformations, A_1 (similar conformation to energy minimized structure), B_1 (with a single phenyl group with a lower dihedral angle with the pyrazinacene) and C_1 (where two phenyl groups at the same end of the molecule have lower dihedral angles). It is likely that this difference in conformational preference between **1** and **2** (i.e., **1** commonly adopts a range of conformations while **2** largely adopts a conformation similar to its crystallographic form plus occasional defects) originates in interactions with the substrate lattice so that **2** is more easily accommodated in its preferred conformation while **1** is required to adapt (through dihedral angle variation) in order to form the line structures. Therefore, the line structures may be the lower energy state favored by both molecules over a dispersed state at the expense of phenyl group dihedral angle variation.

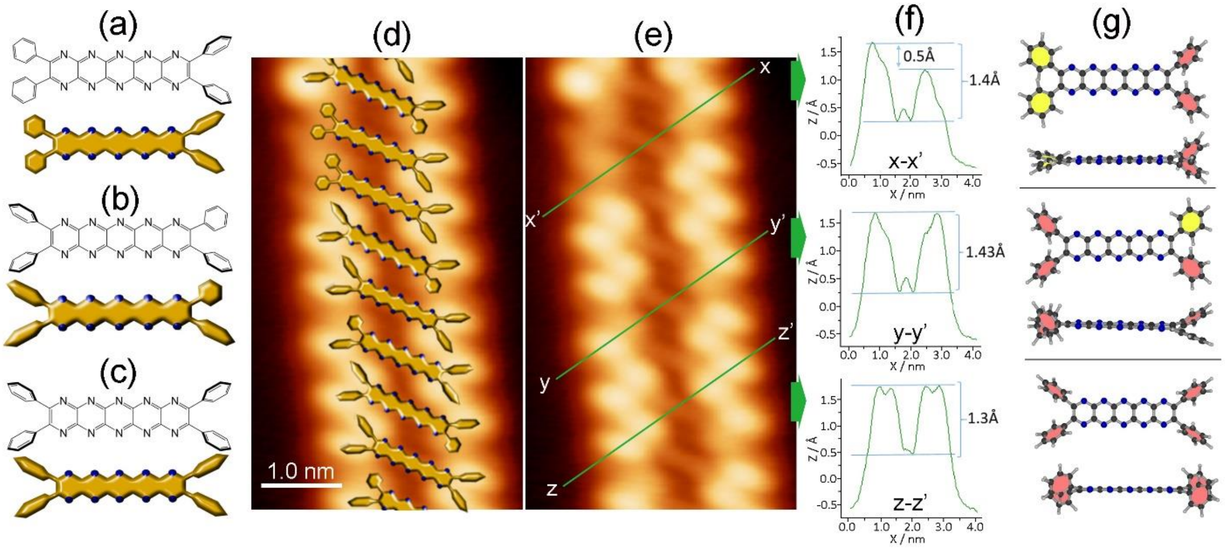

Supplementary Figure 14. Line structures and representations of the relative conformations of phenyl rings contained in line structures of **1**-ox based on estimated dihedral angles between phenyl susbtituents and pyrazinacene backbone. (a) Two phenyl groups: low dihedral angle, two phenyl groups: high dihedral angle. (b) One phenyl group: low dihedral angle, three phenyl groups: high dihedral angle. (c) Two phenyl groups: high dihedral angle. (d) STM image of line structure with each molecule assigned conformations based on (a)-(c). (e) Profiles of line structures revealing height differences related to phenyl group conformation. (f) Corresponding profiles at the lines x-x‘, y-y‘ and z-z‘ drawn in (e). (g) Ball-and-stick structures. Solid pink shading indicates high dihedral angle of phenyl group (> 50°), solid yellow shading indicates low dihedral angle (~10°).

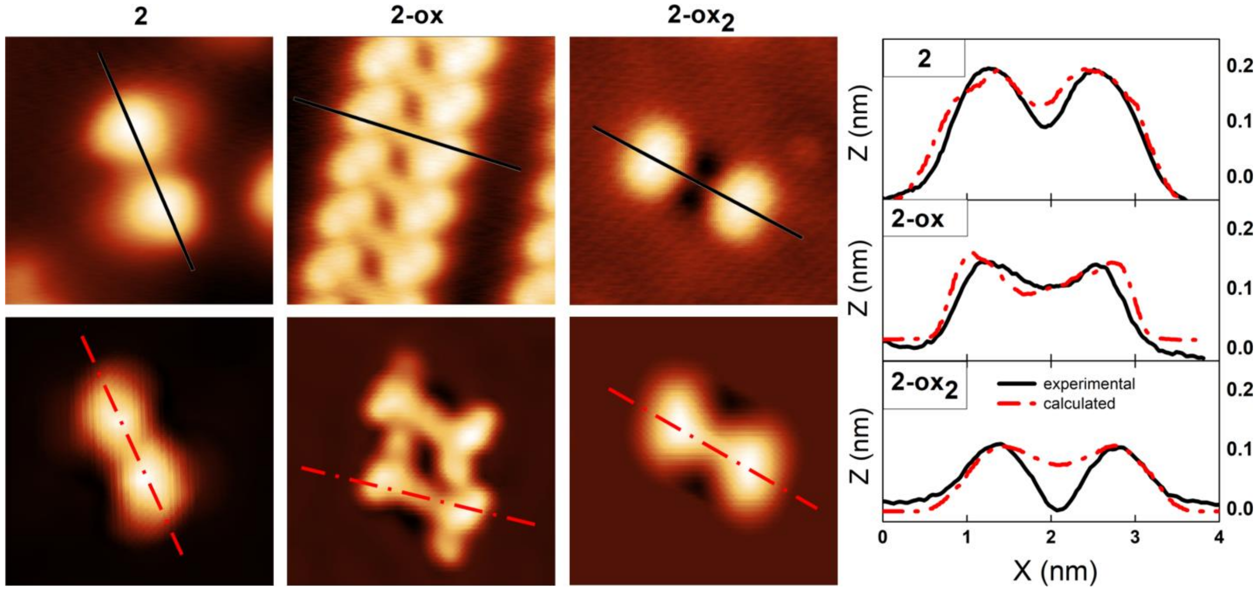

Supplementary Figure 15. DFT Simulation of on-surface structures - STM evolution of **2** after deposition on Cu(111). **Top:** Experimental STM micrographs of **2**, **2**-ox and **2**-ox$_2$. **Bottom:** DFT simulated STM images of **2**, **2**-ox and **2**-ox$_2$. Cross-sectional profiles (at right) have been taken along the lines indicated in (black, experimental STM) and (red-dashed, DFT-simulated STM) as shown at left. Substantial flattening of the molecule upon cyclodehydrogenation to **2**-ox$_2$ is notable. The simulation also reveals the on-surface chirality of **2** caused by its symmetry breaking in consequence of the interaction with Cu(111) (see Fig. 2d and Supplementary Fig. S9), the intermolecular C-H...N hydrogen bonding interaction forming the self-assembled chains of **2**-ox, and the low density of state regions found experimentally at the center of the adsorbed **2**-ox$_2$ molecule as we attribute them to the lone pairs.

	N atom	Vacuum	Adsorbed	Difference<br><br>vacuum - ads
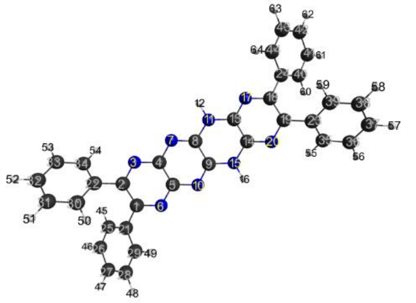	N3	-0.140	-0.137	-0.003
	N6	-0.144	-0.148	+0.004
	N7	-0.171	-0.176	+0.005
	N10	-0.186	-0.188	+0.002
	N11	-0.050	-0.040	-0.010
	N15	-0.049	-0.035	-0.014
	N17	- 0.158	-0.152	-0.006
	N20	-0.154	-0.149	-0.005
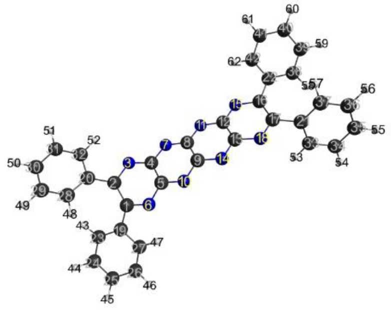	**N3**	-0.129	-0.141	**+0.012**
	N6	-0.125	-0.127	+0.002
	**N7**	-0.139	-0.174	**+0.035**
	N10	-0.129	-0.173	+0.044
	**N11**	-0.139	-0.177	**+0.038**
	N14	-0.135	-0.171	+0.036
	**N15**	- 0.135	- 0.143	**+0.008**
	N18	-0.132	-0.145	+0.013
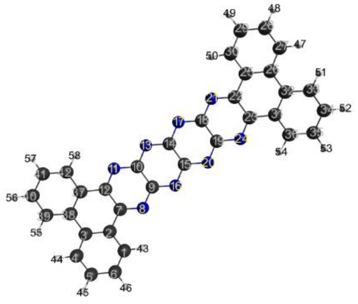	N8	-0.124	-0.158	+0.034
	N11	-0.115	-0.148	+0.033
	N13	-0.126	-0.192	+0.066
	N16	-0.128	-0.193	+0.065
	N17	-0.129	-0.184	+0.055
	N20	-0.126	-0.182	+0.056
	N21	- 0.121	-0.165	+0.044
	N24	-0.120	-0.149	+0.029

Supplementary Figure 16: Charge characteristics of **2**, **2**-ox and **2**-ox$_2$ on the Cu(III) surface showing Voronoi electronic population on N atoms in compound **2**. Top: **2**, middle: **2**-ox; bottom: **2**-ox$_2$. For compound **2**-ox N atoms that approach closer to the surface during the adsorption process are indicated in **bold**). The numbers represent the total net charge of the atom: the variation from the neutral charge, in units of |e|: positive (negative) values indicate deficiency (excess) of electrons in the atom.

Supplementary Note 2. To discuss the energies of the lines in the XPS N1s spectra we have analyzed the electronic populations of the adsorbed molecules. The Voronoi charges[S1,S2] gained/lost for each atom are given in Supplementary Fig S16 above. In particular, it can be seen that for **2**-ox and **2**-ox$_2$ the Voronoi populations are not related to the average distance between the nitrogens and the surface. Indeed, the geometry of the **2**-ox system indicates that the nitrogens on one side of the central part of the molecule are closer to the surface than those on the other side. The average value for the Voronoi population, however, is -0.16 in both cases (i.e. left and right side of the central π system). For the **2**-ox$_2$ system, the average value is very close to that obtained in the case of **2**-ox, i.e. -0.17 e. While the average values are

the same in both cases, differences up to 0.04 e may occur between specific atoms, most probably caused by the local geometry: the distance between a given nitrogen and the closest Cu atom will specifically influence its electronic population. Indeed, in vacuum these differences amount to less than 0.01 e in most cases. For **2**. we see that N bonded to H atoms exhibits population around -0.04 e, while for the other nitrogens exhibiting a free electron pair, the average population is -0.16 e. This agrees favorably with the observation that only the native molecules **1** and **2** exhibit two peaks. The first lower intensity signal is due to nitrogens bonded to N-H (corresponding to a population of -0.04 e) with a second one specific to other pyridine-type N. This peak is rather similar in the Voronoi charge argument regardless of the position of the N atom within the molecule and whether it is dehydrogenated or cyclodehydrogenated. This peak corresponds to an average electron population of -0.16 to -0.17 e. All this reasoning supports the experimental assignment that the molecules **1** and **2** are physisorbed, not chemisorbed, to the substrate. (Supplementary Information Pages S19-S20).

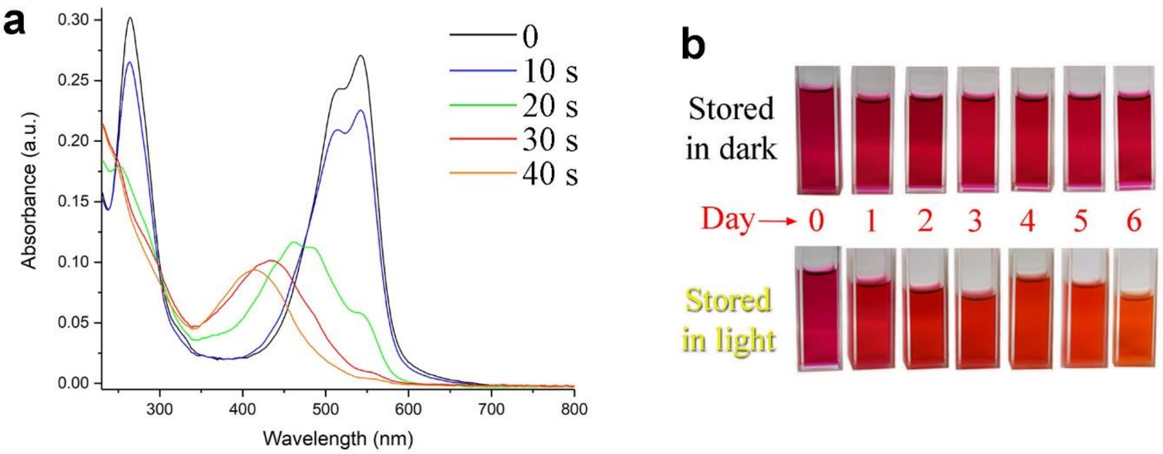

Supplementary Figure 17. Photoreduction of **2**. **a**, Electronic absorption spectra of **2** (in dichloromethane) taken at 10 second intervals during irradiation with a high intensity UV lamp. At 0 s and 10 s, **2**-ox is largely present with **2** predominating after 20 s. Beyond 30 s a shift in absorbance maximum may be due to decomposition to an unidentified product. Reducing intensity of the absorption maximum also suggests decomposition, which is in contrast to **1** where isobesticity is observed for this process. **b**, Photographs of solutions of **2**-ox stored in the dark (upper) or stored under room light (lower) taken at 1 day intervals revealing a gradual reappearance of the orange hue due to **2** in solution stored in light.

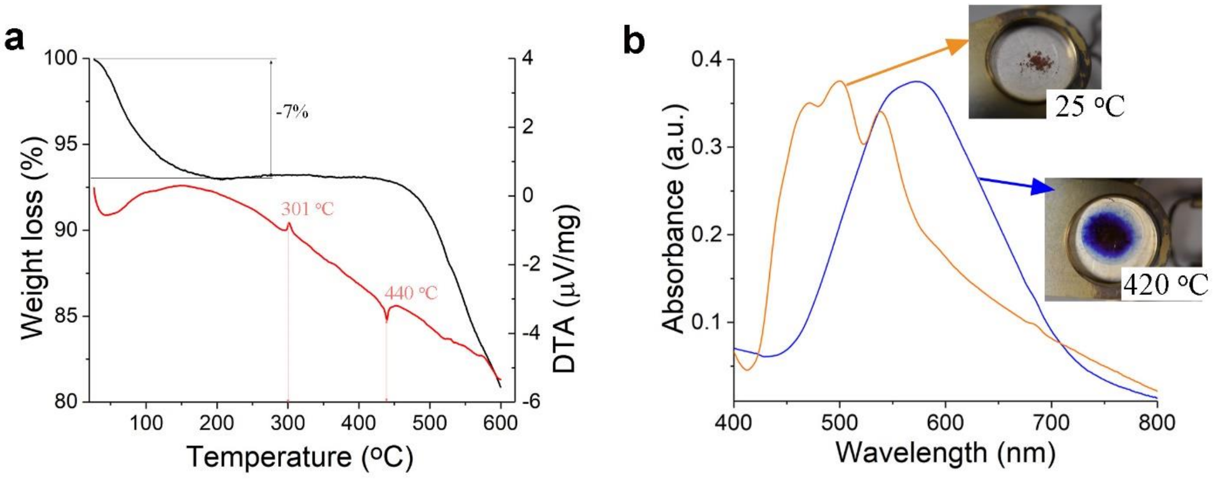

Supplementary Figure 18. Thermal analyses of **1**. **a**, Thermogravimetric analysis. Weight loss of 7% from room temperature corresponds well with loss of 2.5 mols of water (expected from elemental analysis – see synthesis section). Peaks in the DTA trace correspond to a phase transition (301 °C, probably melting) and commencement of decomposition or dihydropyrazine dehydrogenation (440 °C). The latter is signified by appearance of the deep blue colour due to the decaazapentacene chromophore – see **b**. **b**, Solid state electronic absorption spectra of **1** at room temperature (orange line) and after heating in air at 420 °C for 10 minutes.

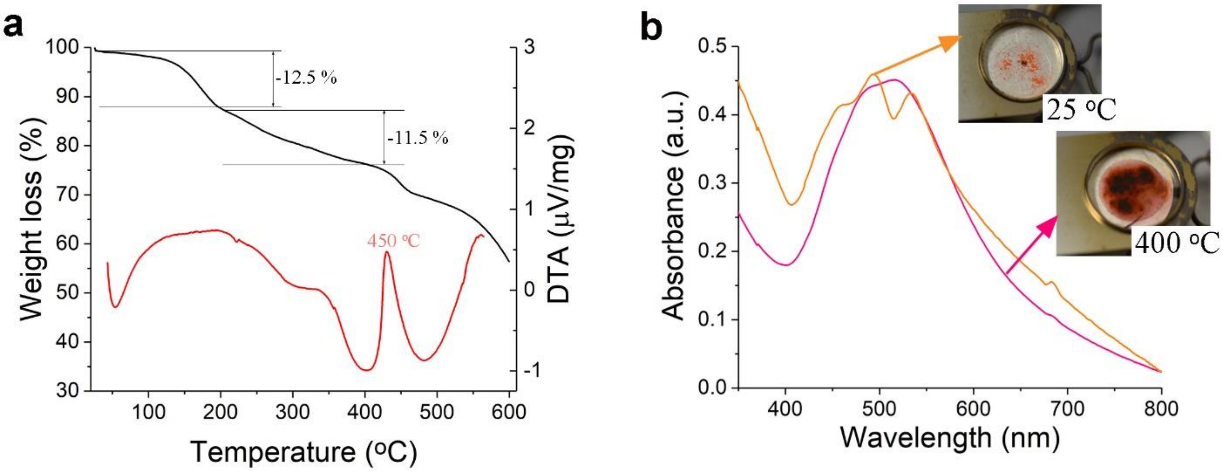

Supplementary Figure 19. Thermal analyses of **2**. **a**, Thermogravimetric analysis. Total loss of volatiles at low temperatures corresponds well with that found by elemental analysis (~25 %). Decomposition commences at ~400 °C. The gradual loss of weight up to decomposition of this sample is due to reactions involving decomposition of tetrahydrofuran solvent of crystallization. **b**, Solid state electronic absorption spectra of **2** at room temperature (orange line) and after heating in air at 400 °C for 10 minutes. There is a ~50 nm red shift in the absorption maximum of **2** in the solid state.

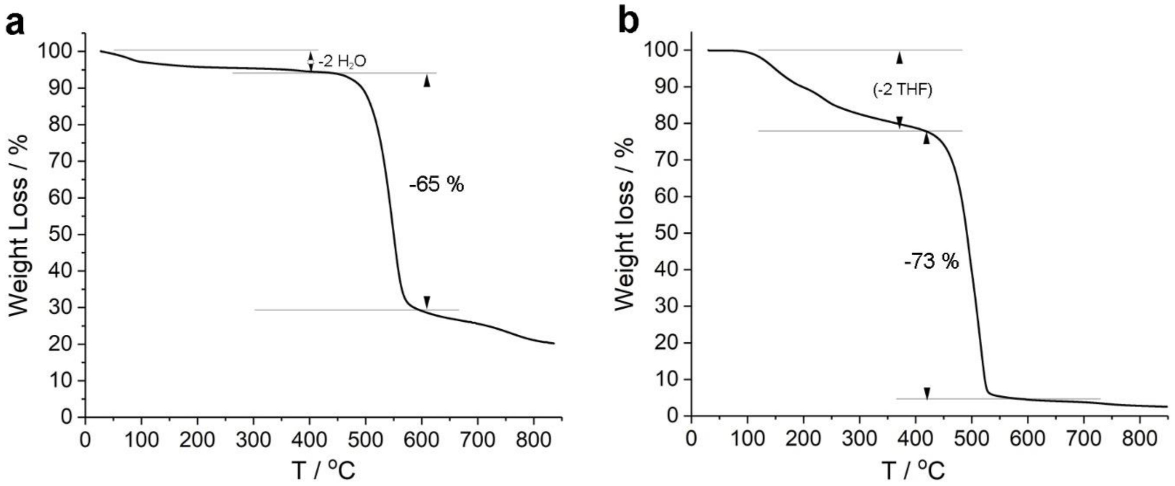

Supplementary Figure 20. Additional thermogravimetric analysis (TGA). **a**, TGA of **1**. Weight loss of 7% from room temperature corresponds well with loss of 2.5 mols of water (expected from elemental analysis – see synthesis section). Peaks in the DTA trace correspond to a phase transition (301 °C, probably melting) and commencement of decomposition or dihydropyrazine dehydrogenation (440 °C). **b**, TGA of **2**. Total loss of volatiles at low temperatures corresponds well with that found by elemental analysis (~25 %). Decomposition commences at ~400 °C. The gradual loss of weight up to decomposition of this sample is due to reactions involving decomposition of tetrahydrofuran solvent of crystallization. Note that **2** also sublimes close to its decomposition temperature during TGA measurements so that the remnant mass is low.

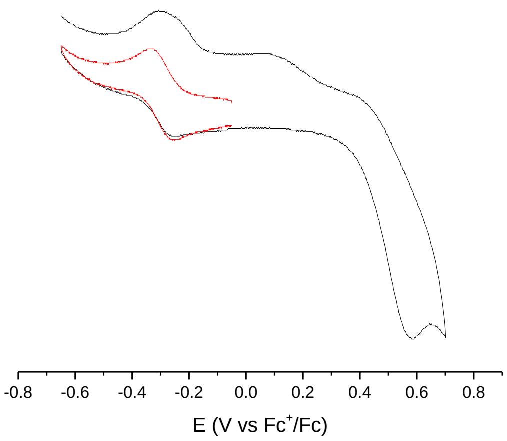

Supplementary Figure 21. Cyclic voltammetry for **2**-ox revealing a first reduction at -0.293 V (in $CHCl_3$/0.2 M tetrabutylammonium perchlorate; scan rate: 100 mV s^{-1}). **1**-ox could not be obtained in a form suitable for electrochemical measurements due to its poor solubility.

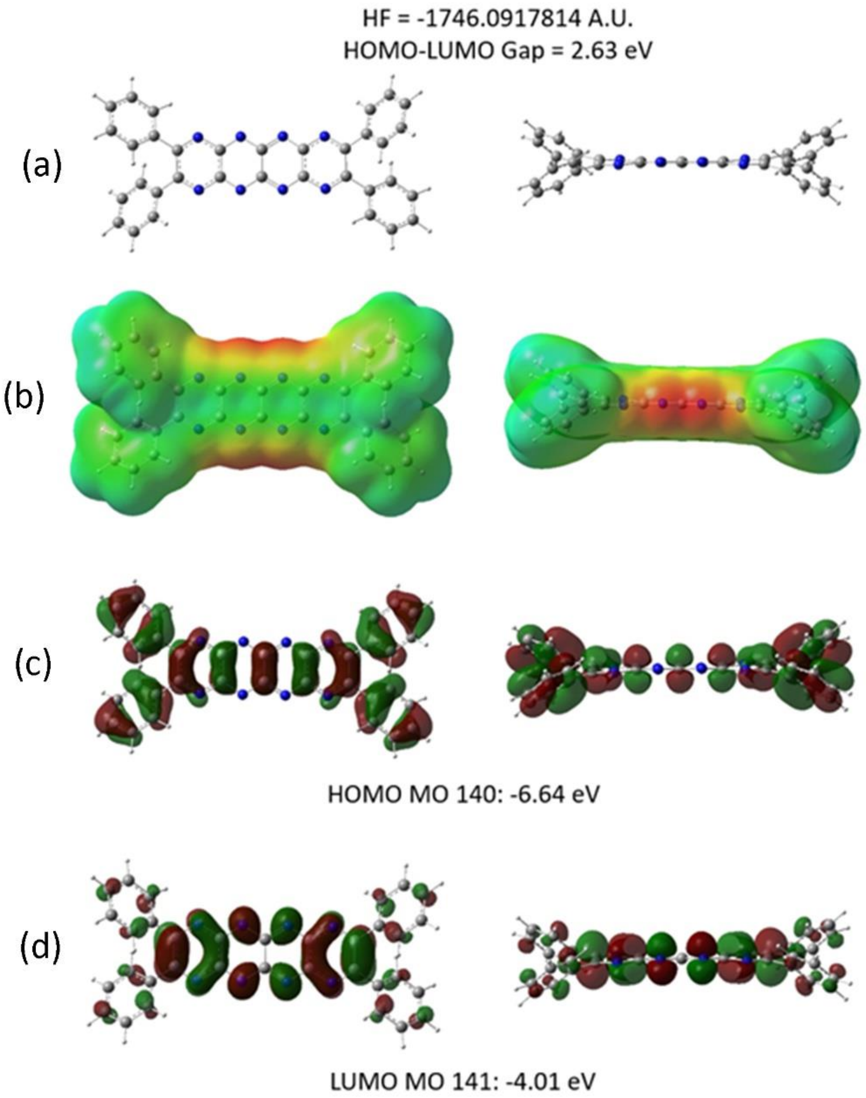

Supplementary Figure 22. DFT calculations for compound **2**-ox. (a) Molecular structure. (b) Surface charge. (c) Highest occupied molecular orbital (HOMO), and (d) lowest unoccupied molecular orbital (LUMO).

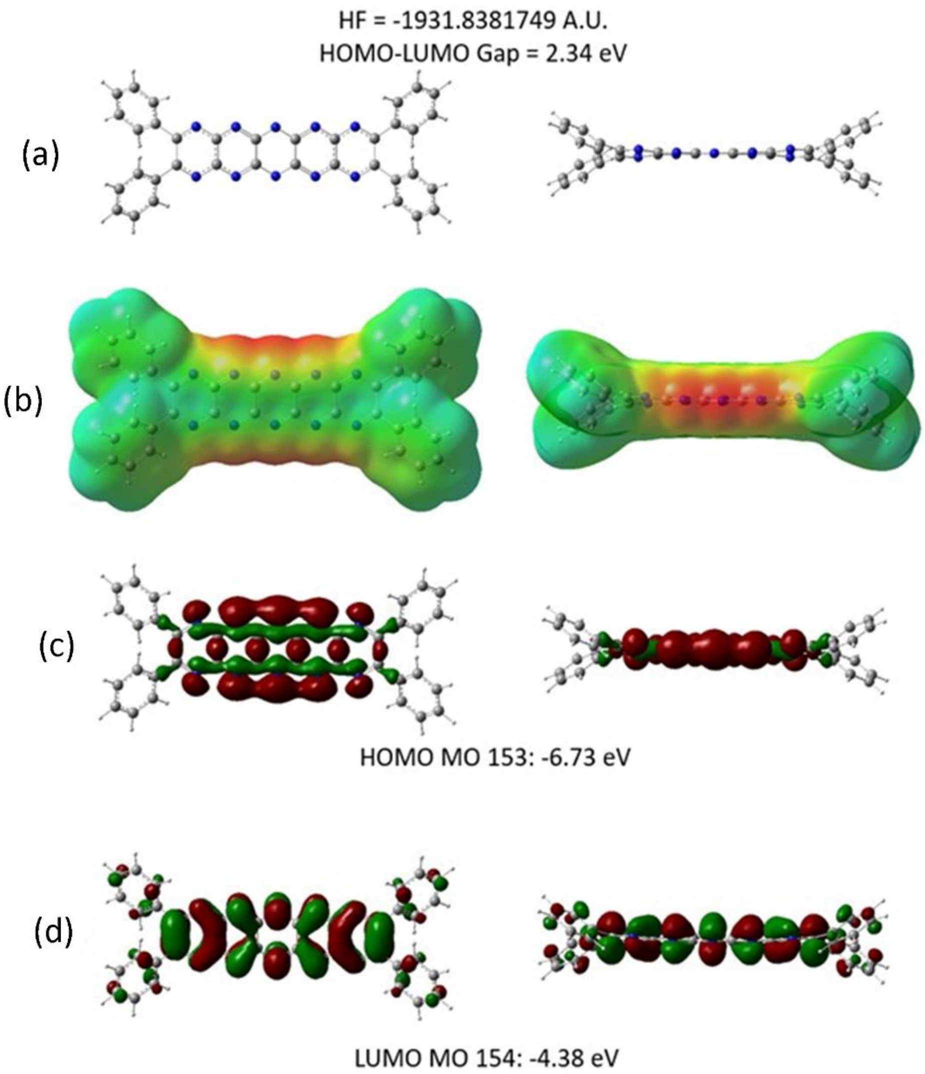

Supplementary Figure 23. DFT calculations for compound **1**-ox. (a) Molecular structure. (b) Surface charge. (c) Highest occupied molecular orbital (HOMO), and (d) lowest unoccupied molecular orbital (LUMO).

Supplementary Note 3. In view of the emerging importance in many domains of chemical science and technology, in particular, for light harvesting, we have also investigated the photoredox catalysis of these novel azaacenes. The photo-induced reduction of **1**-ox and **2**-ox, their on-surface reactivity and their reactivity in solution (i.e. by the step-wise dehydrogenation of the pyrazinacene core on the surface and electrochemically) indicate that these compounds are also of interest from a photoredox point-of-view. Note that their optical and redox properties in solution also resemble those recently reported for a push-pull dicyanopyrazine[S3-S7] and a similar tetraazatetracene[S8]. For these reasons, we examined the photoredox catalytic activities in the benchmark cross-dehydrogenative coupling (CDC) reaction[S9] of **1** and **2** between *N*-phenyltetrahydroisoquinoline (THIQ) and nitromethane as shown in Supplementary Scheme S1 (also Supplementary Table S1 – see below). The reactions were run for 24 hours with 1 mol% of the catalyst by irradiating the reaction mixture with light emitted by a Royal Blue LED under air at 25 °C. Both pyrazinacenes were capable of causing C-C bond formation between THIQ and nitromethane. The catalytic performances of **1** and **2**, however, differ slightly (see Supplementary Table S1). According to the observed conversions and isolated yields, octaazatetracene **2** proved to be a more efficient catalyst than decaazapentacene **1**. Due to the greater compatibility of the absorption maximum of **2** (~450 nm) with the high energy emission band of the Royal Blue LED (~450 nm), **2** afforded better conversion (72 %) and isolated yield (70 %) of the CDC product than **1** (67% and 63 %, resp.). Note that **1** compared to **2** possesses an absorption maximum red-shifted by ~50 nm. (Fig. 5a, main manuscript).

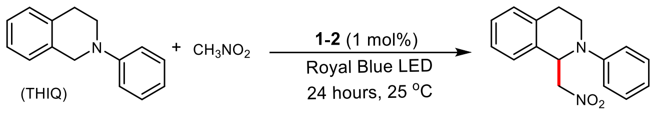

Supplementary Scheme 1. Benchmark CDC reaction.

Supplementary Table 1. Yields of reaction shown in Scheme S1 catalyzed by **1** or **2**.

Comp.	Conversion [%][a]	Isolated yield [%]
**none**	0	0
**1**	67	63
**2**	72	70

a) Calculated from the NMR spectrum of the crude reaction mixture

^{1}H and ^{13}C Nuclear Magnetic Resonance Spectra and Mass Spectra

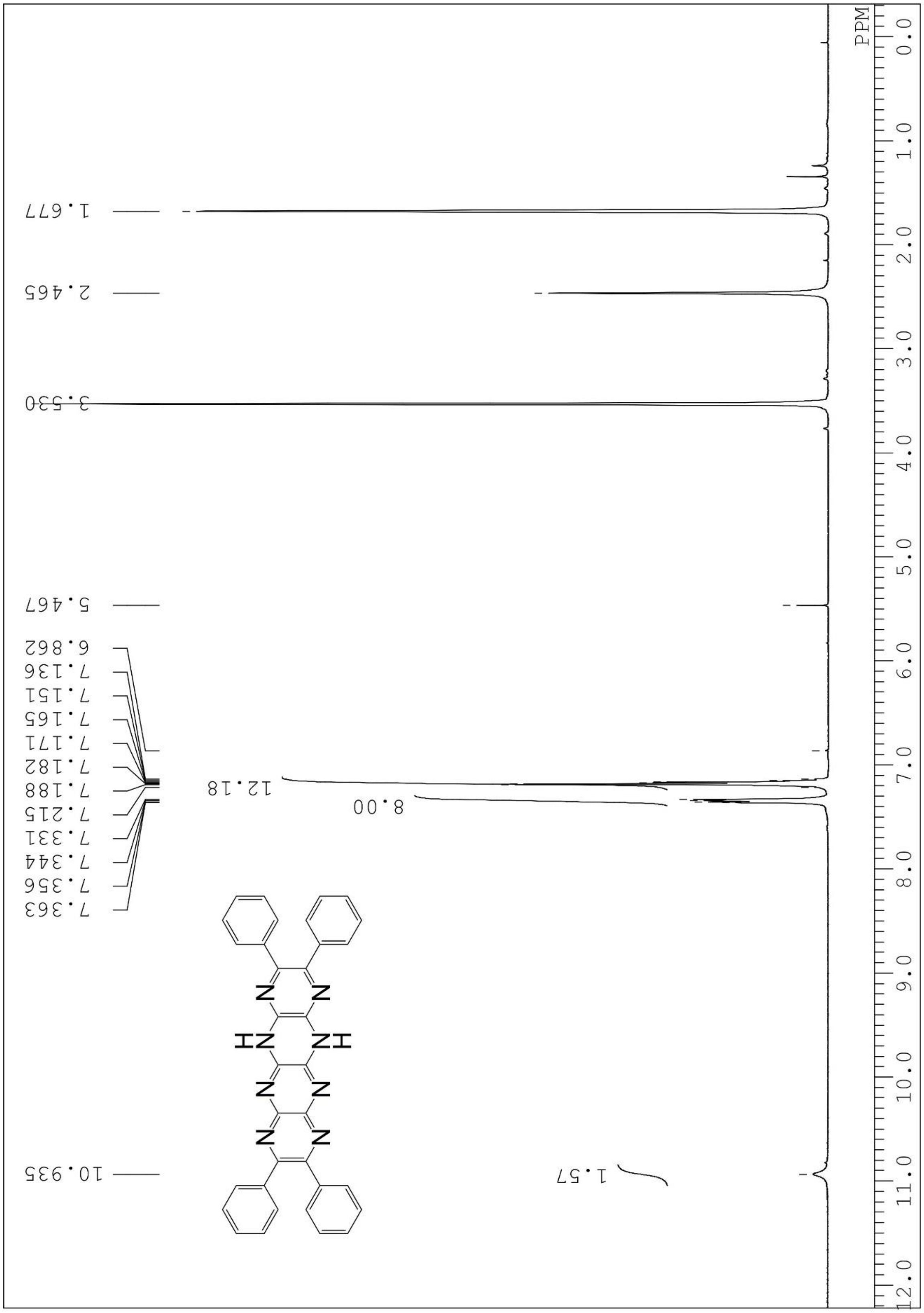

Supplementary Figure 24. ^{1}H-NMR spectrum of **2** in tetrahydrofuran-d_8.

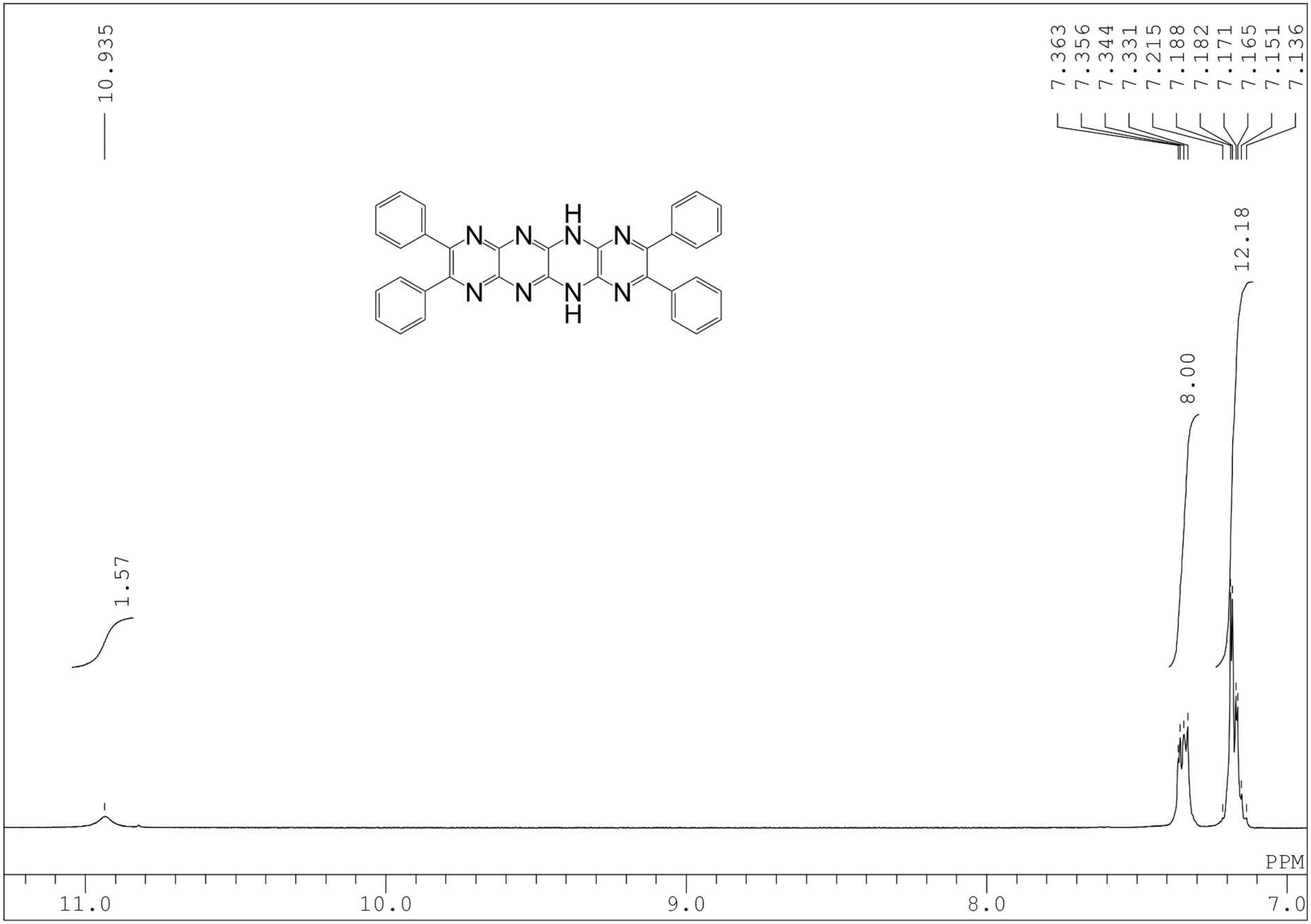

Supplementary Figure 25. Low field region of ^{1}H-NMR spectrum of **2** in tetrahydrofuran-d_8.

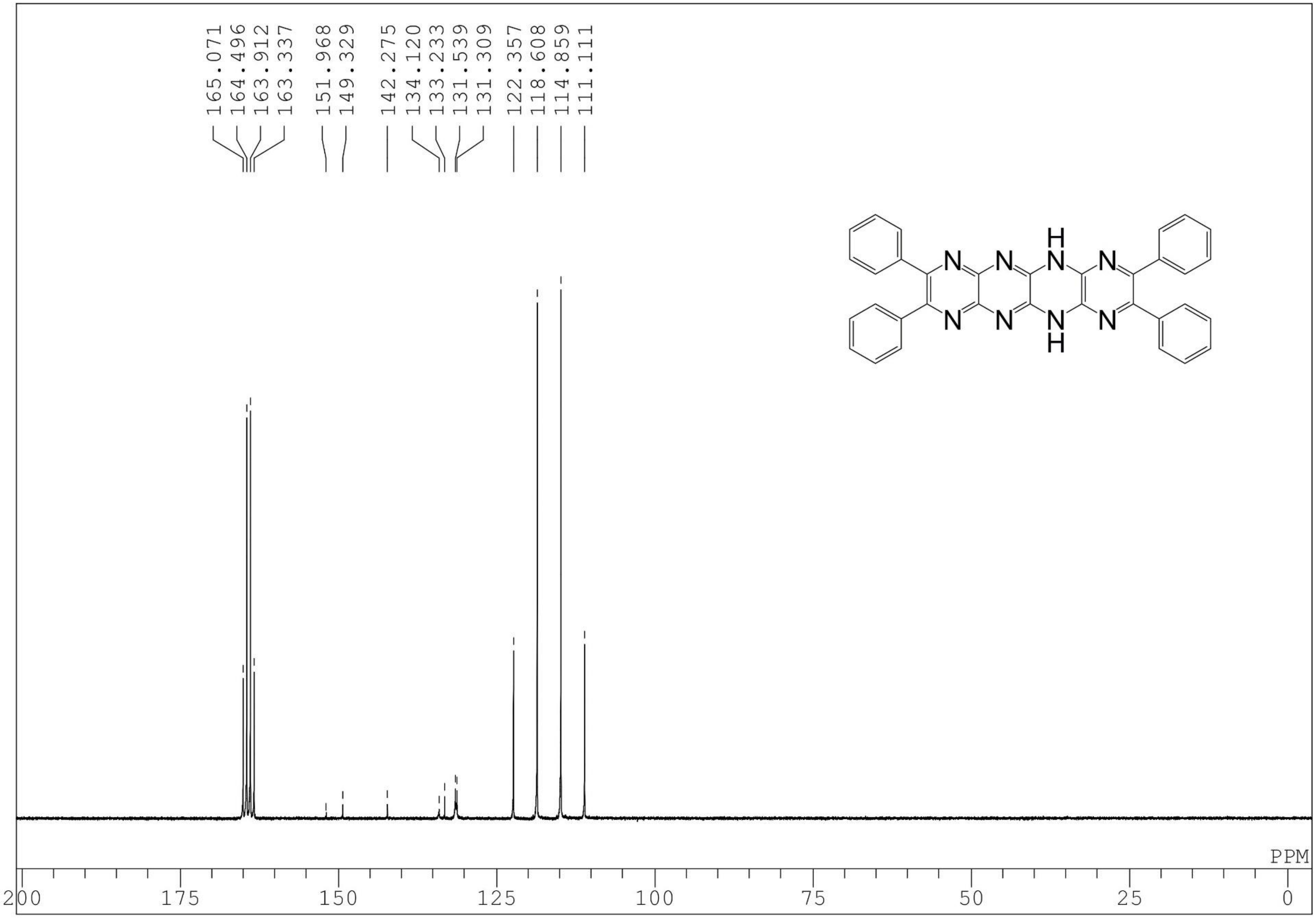

Supplementary Figure 26. ^{13}C-NMR spectrum of **2** in trifluoroacetic acid-d_1.

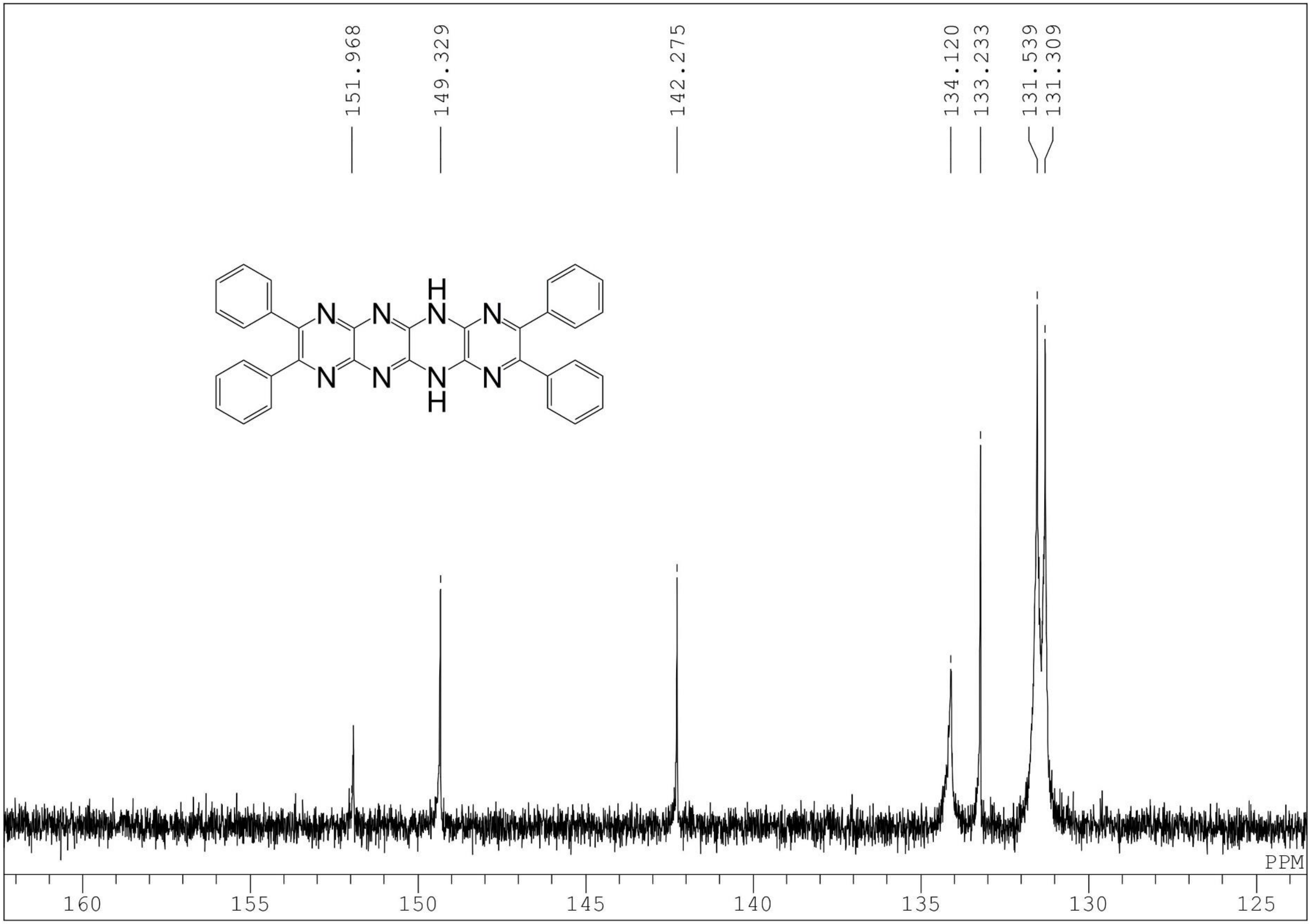

Supplementary Figure 27. Aromatic region of ^{13}C-NMR spectrum of **2** in trifluoroacetic acid-d_1.

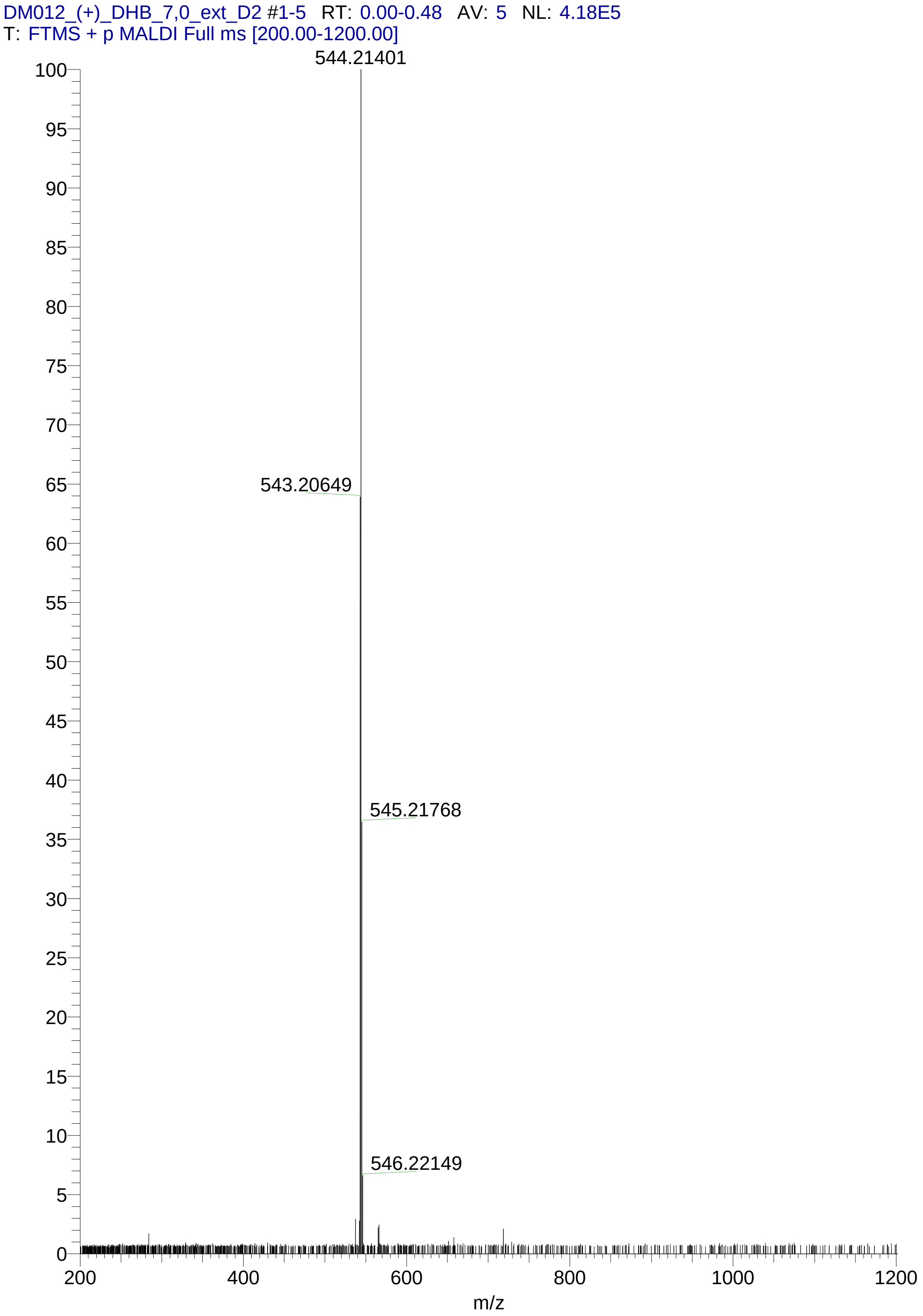

Supplementary Figure 28. High resolution MALDI-TOF mass spectrum of **2**.

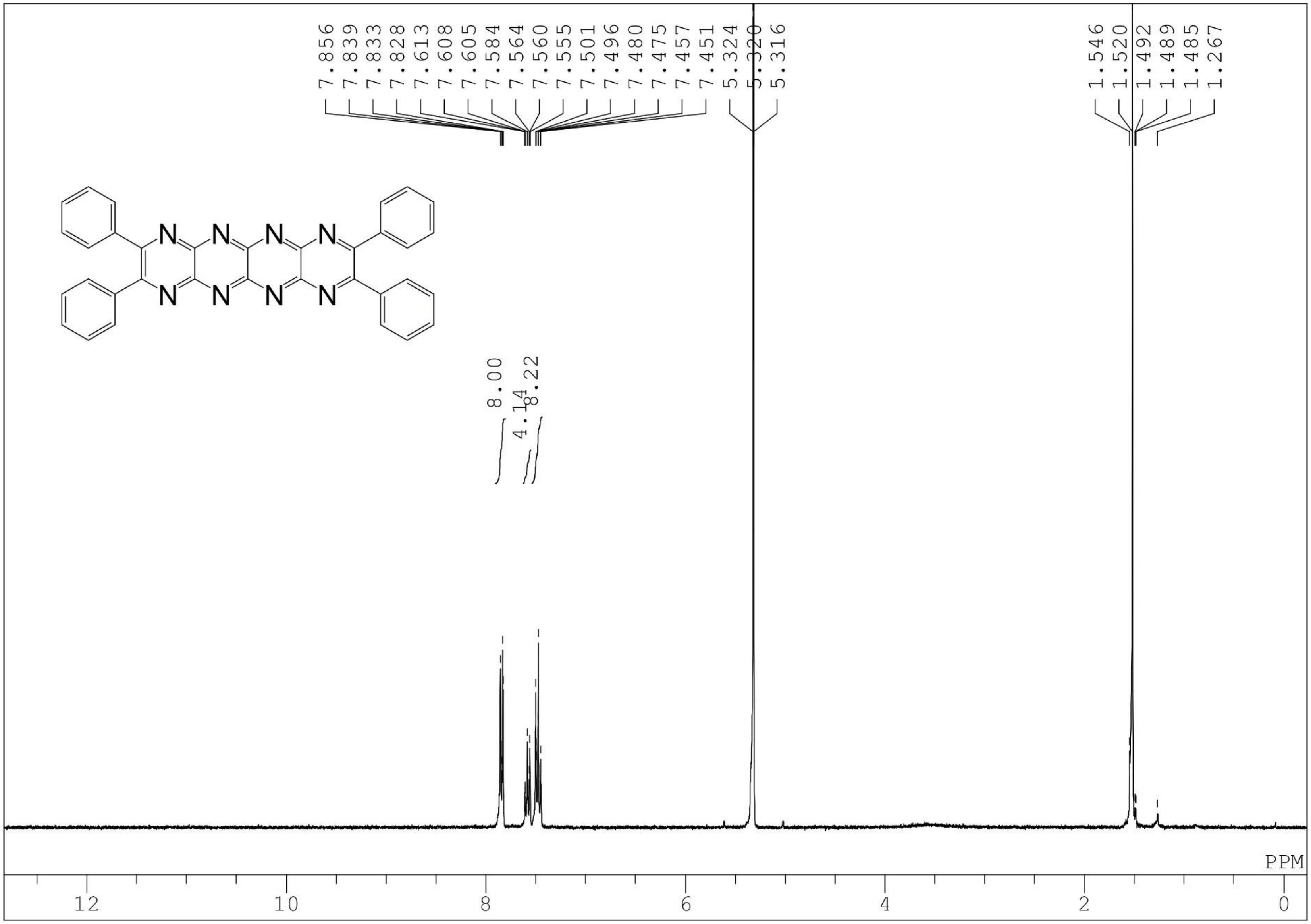

Supplementary Figure 29. ^{1}H-NMR spectrum of **2**-ox in CD_2Cl_2.

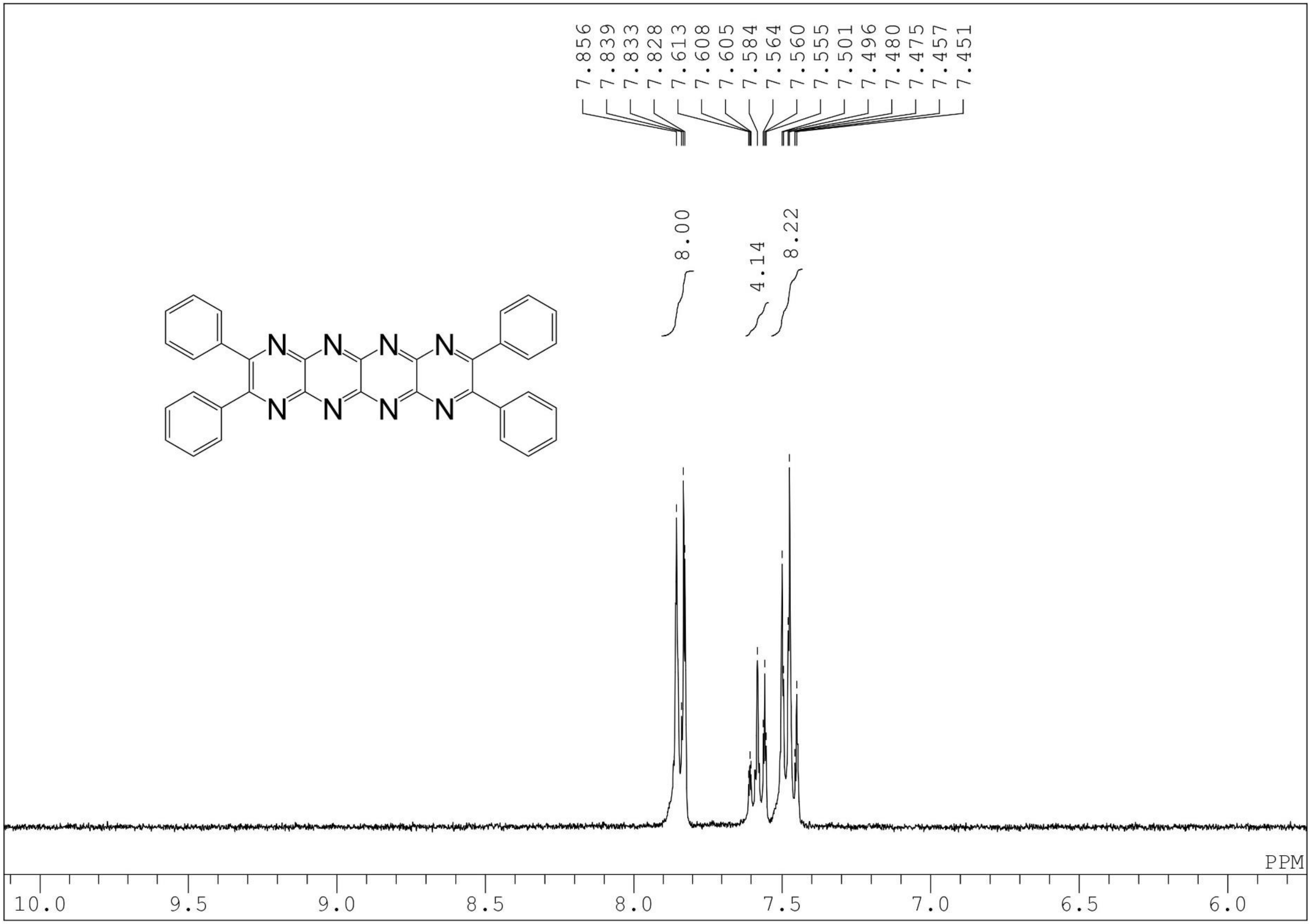

Supplementary Figure 30. Aromatic region of ^{1}H-NMR spectrum of **2**-ox in CD_2Cl_2.

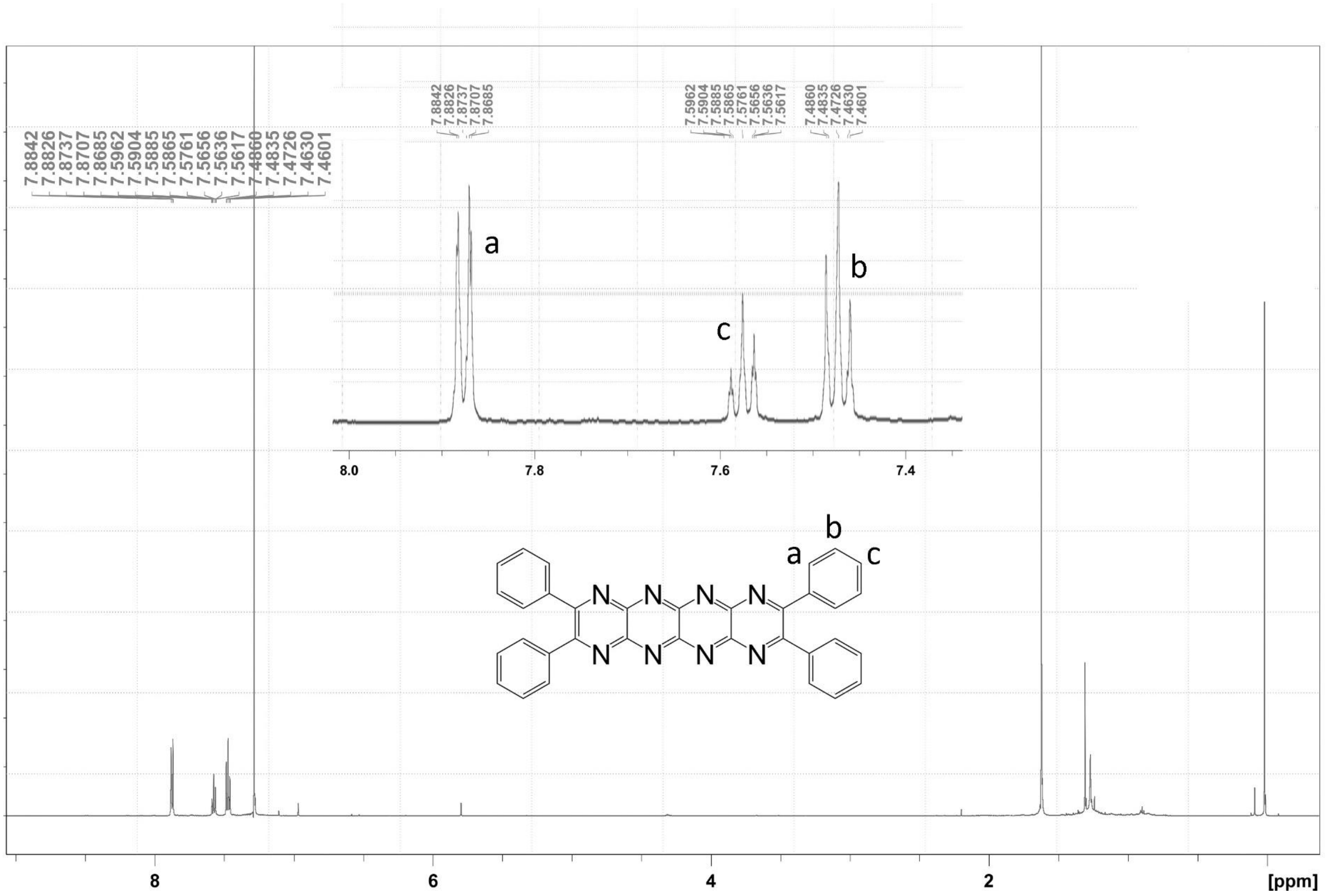

Supplementary Figure 31. ^{1}H-NMR spectrum of **2**-ox in $CDCl_3$. Chemical shifts of phenyl-H are similar to those in CD_2Cl_2.

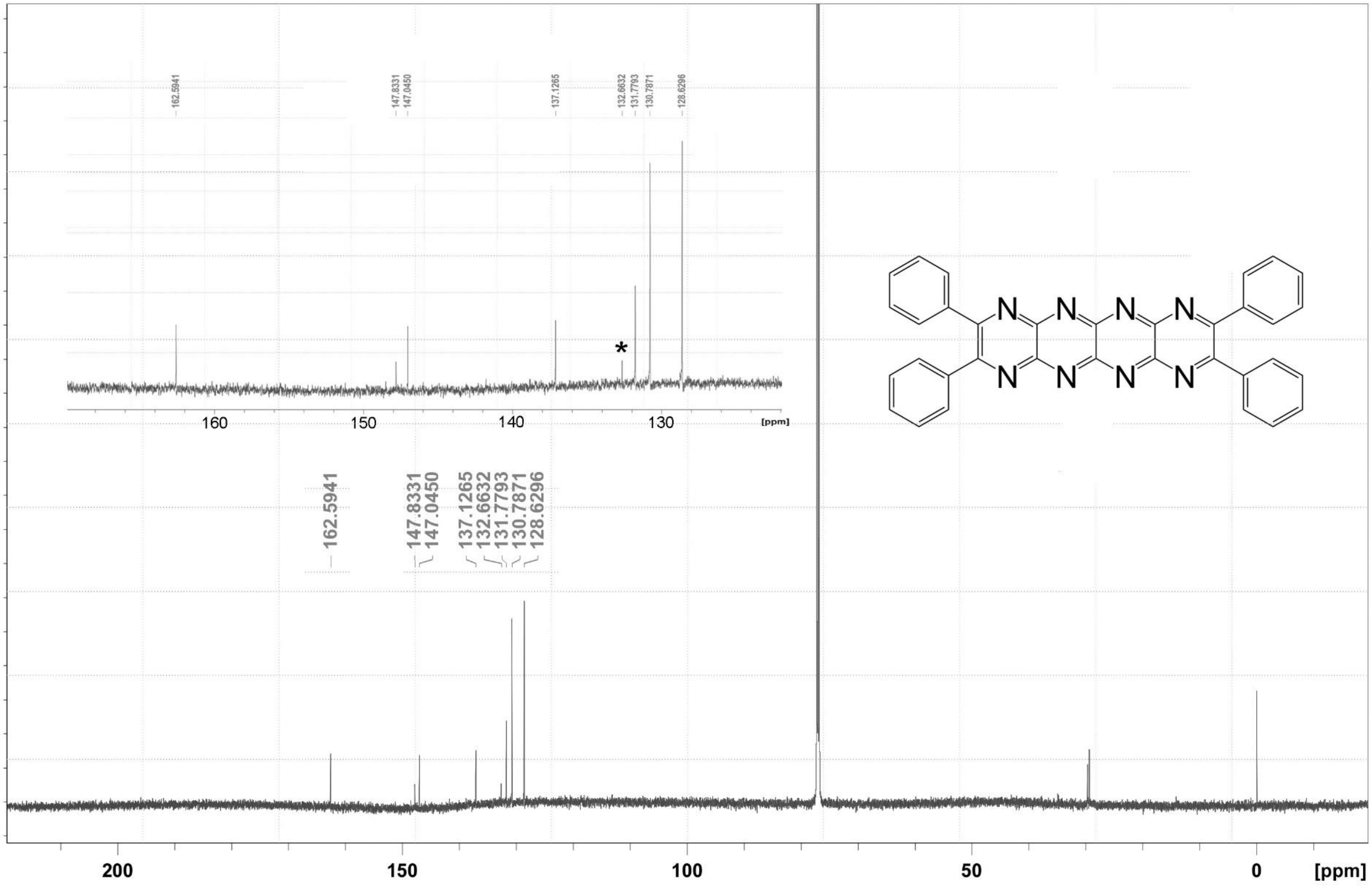

Supplementary Figure 32. ^{13}C-NMR spectrum of **2**-ox in $CDCl_3$. Asterisk denotes impurity – probably compound **2**.

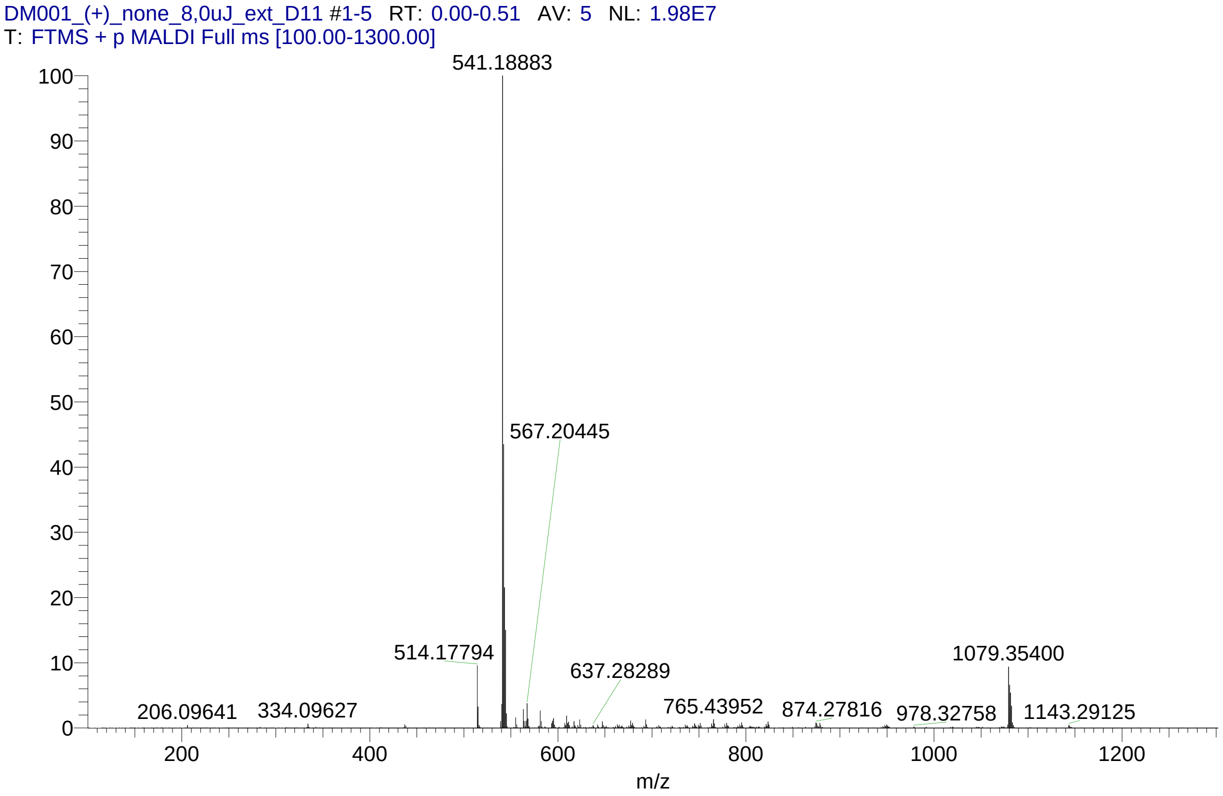

Supplementary Figure 33. High resolution MALDI-TOF mass spectrum of **2**-ox.

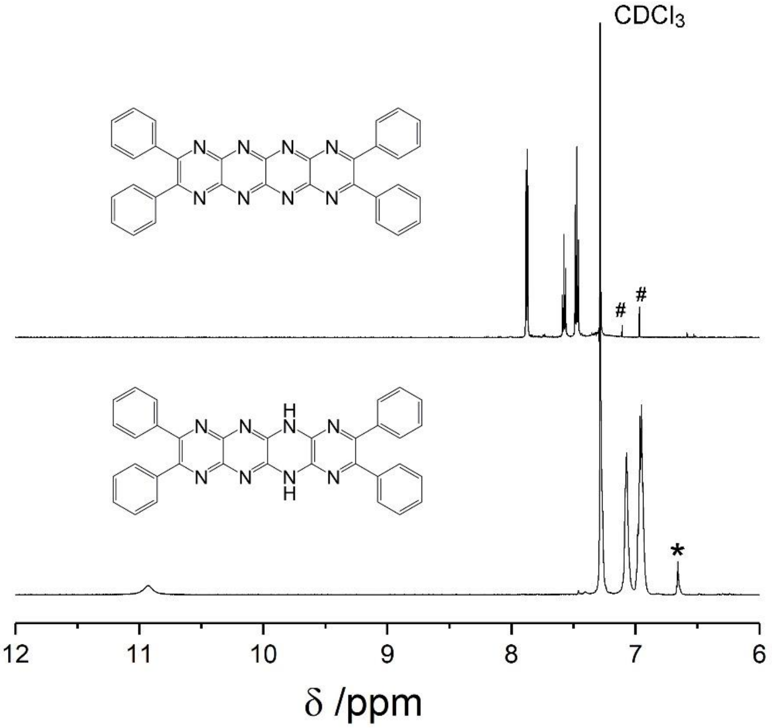

Supplementary Figure 34. ^{1}H-NMR spectra of **2** (lower) and **2**-ox (upper) both in $CDCl_3$ illustrating the shift to lower field of the phenyl aromatic protons and disappearance of the NH resonance following oxidation with PbO_2. Asterisk denotes impurity. # denotes trace of **2**.

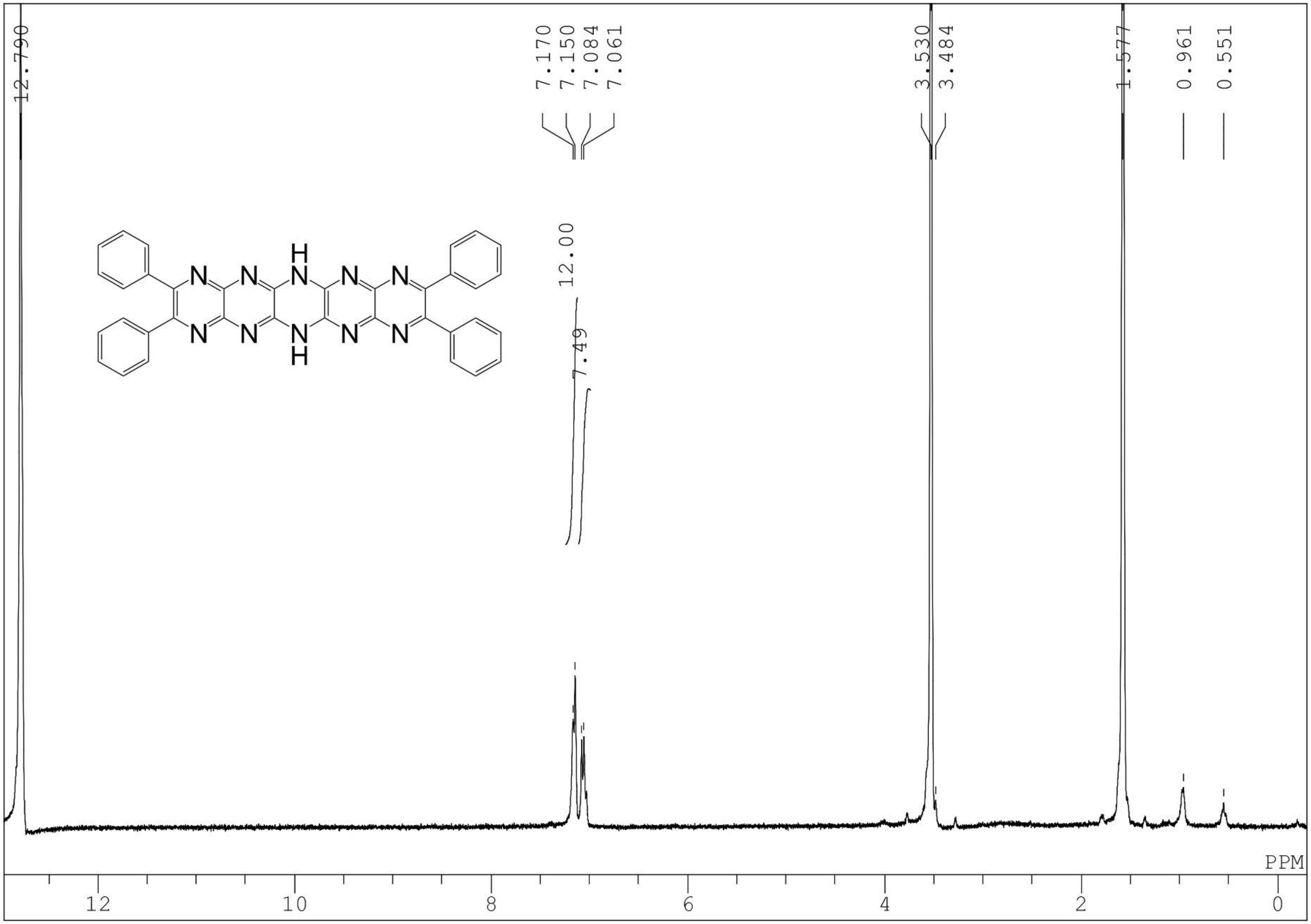

Supplementary Figure 35. ^{1}H-NMR spectrum of **1** in tetrahydrofuran-d_8 with several drops of trifluoroacetic acid-d_1.

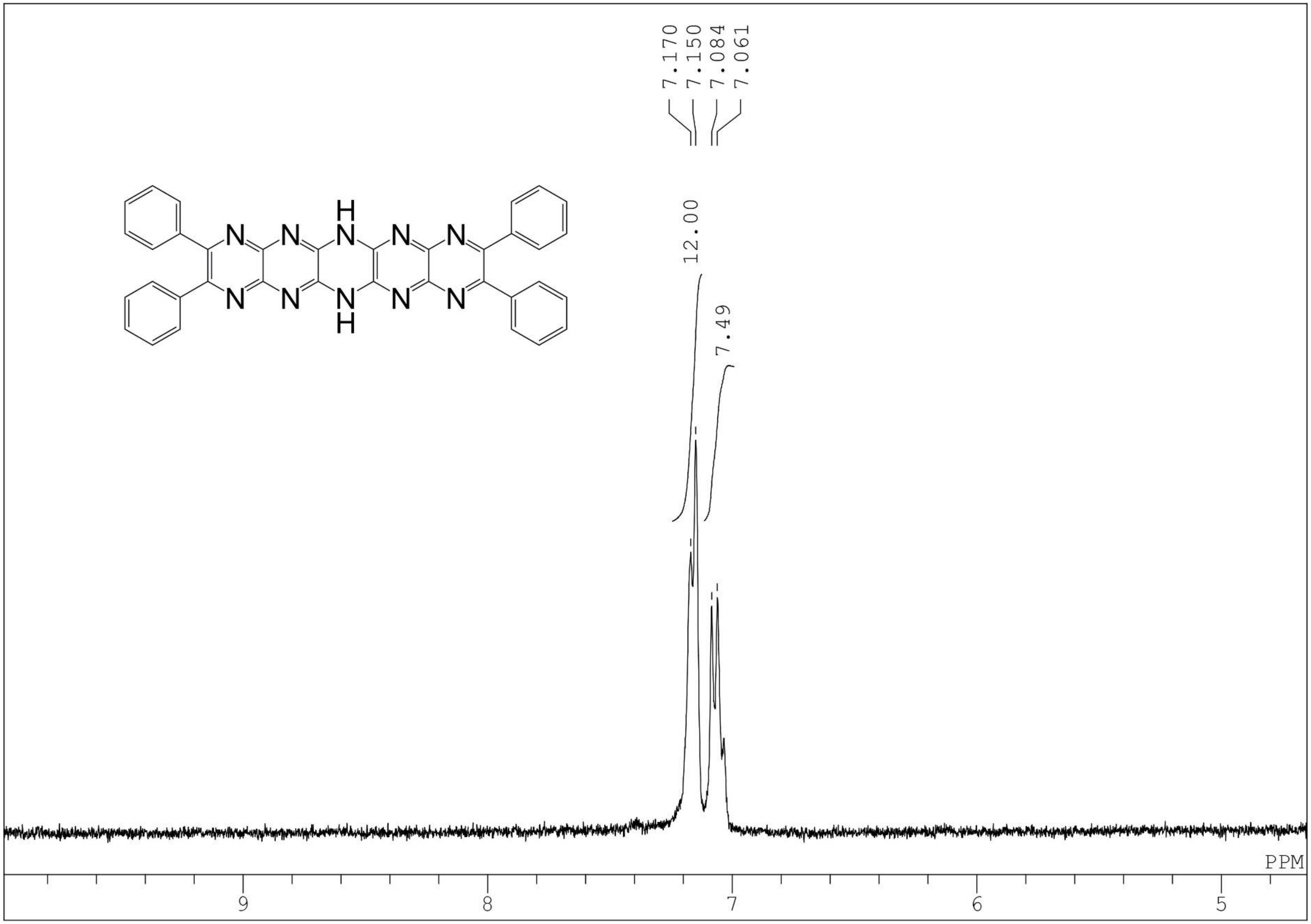

Supplementary Figure 36. Aromatic region of ^{1}H-NMR spectrum of **1** in tetrahydrofuran-d_8 with several drops of trifluoroacetic acid-d_1.

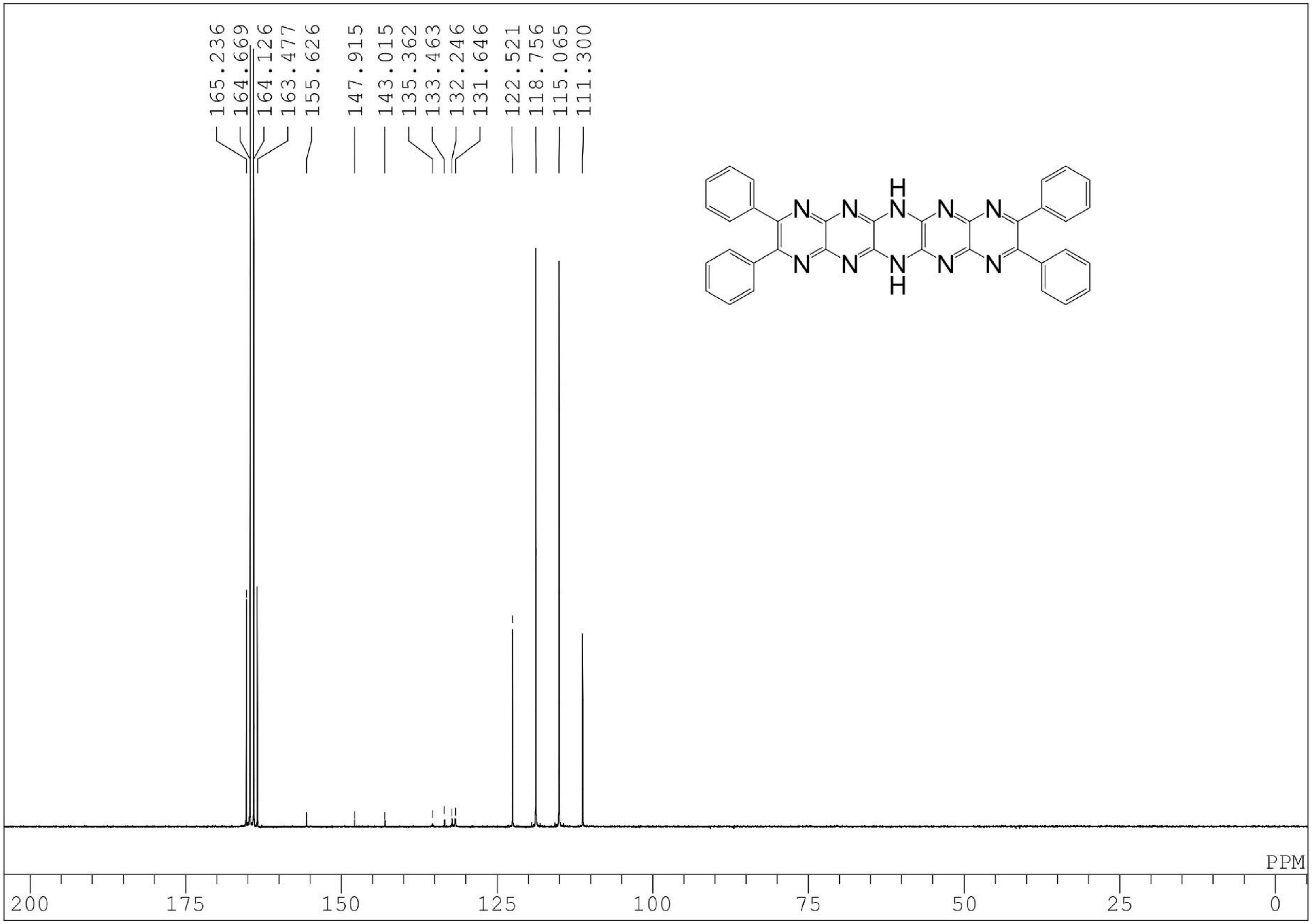

Supplementary Figure 37. ^{13}C-NMR spectrum of **1** in trifluoroacetic acid-d_1.

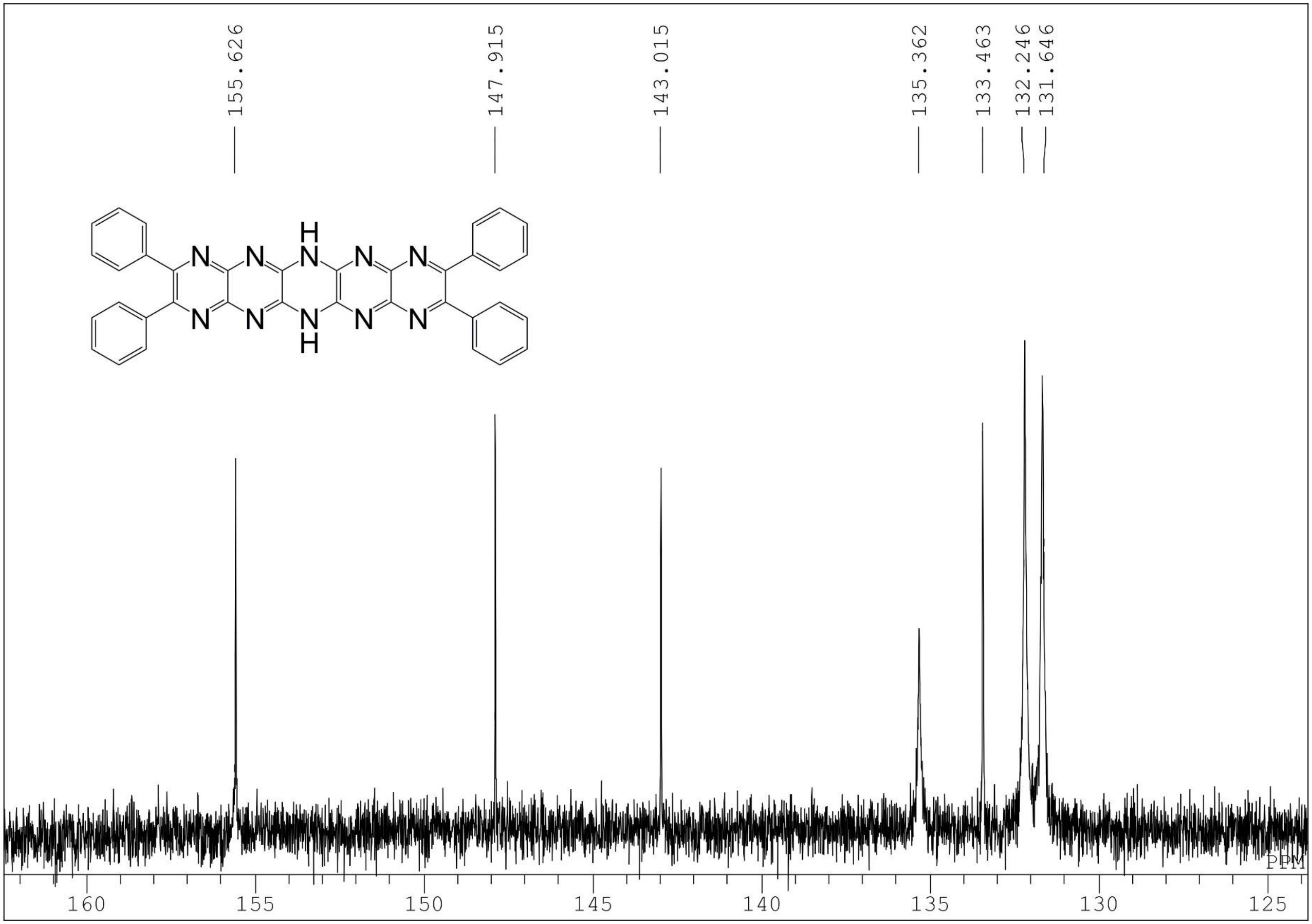

Supplementary Figure 38. Aromatic region of ^{13}C-NMR spectrum of **1** in trifluoroacetic acid-d_1.

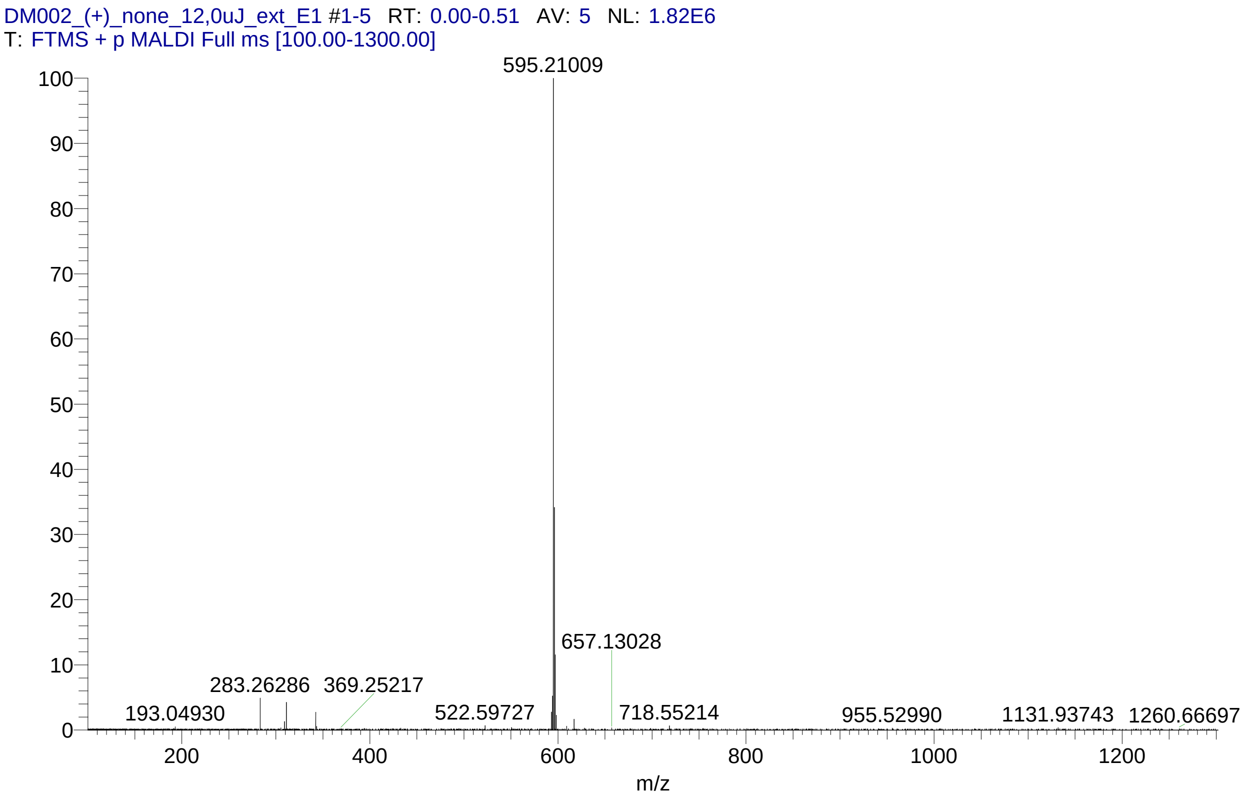

Supplementary Figure 39. High resolution MALDI-TOF mass spectrum of **1**.

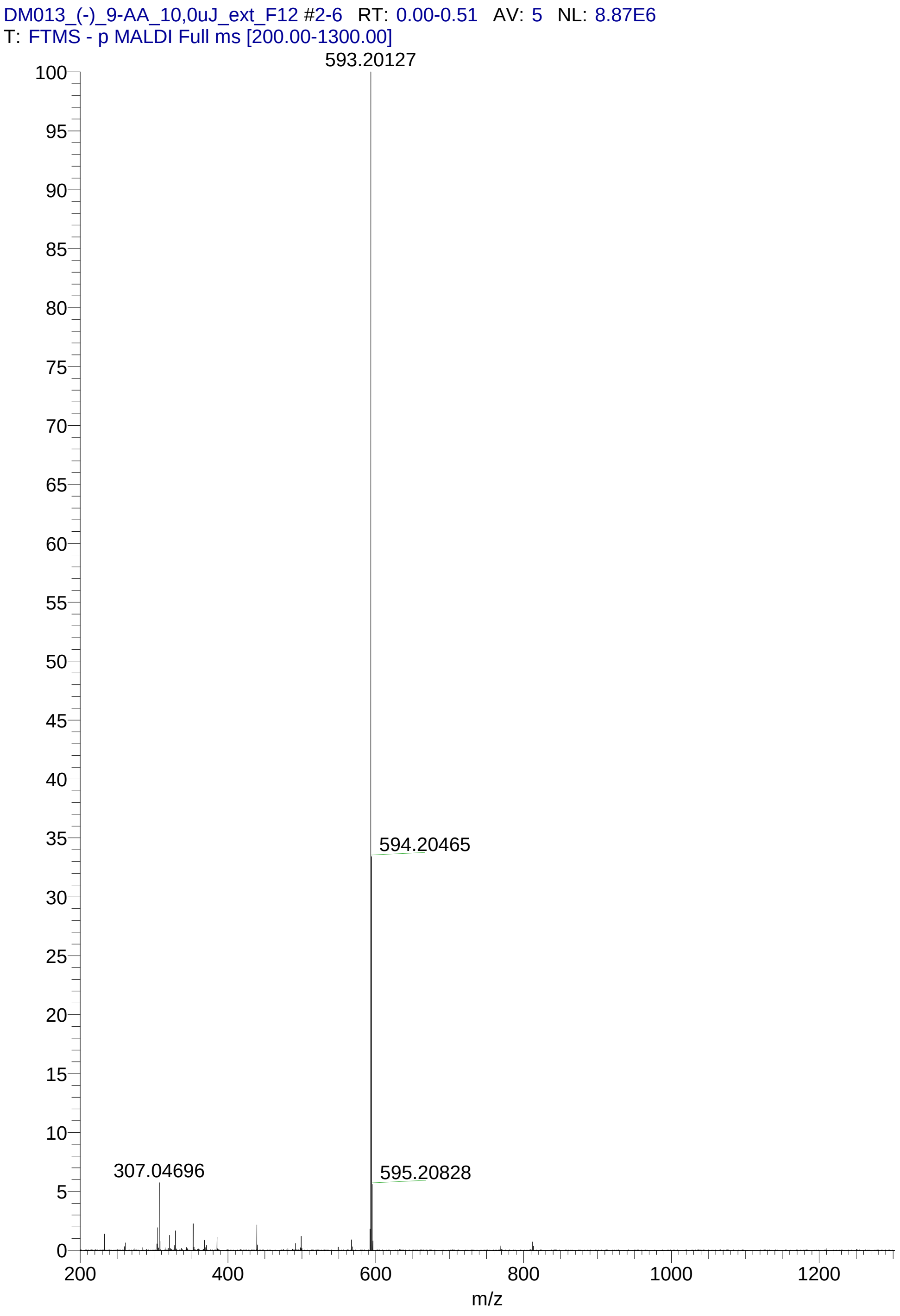

Supplementary Figure 40. High resolution MALDI-TOF mass spectrum of **1**-ox.

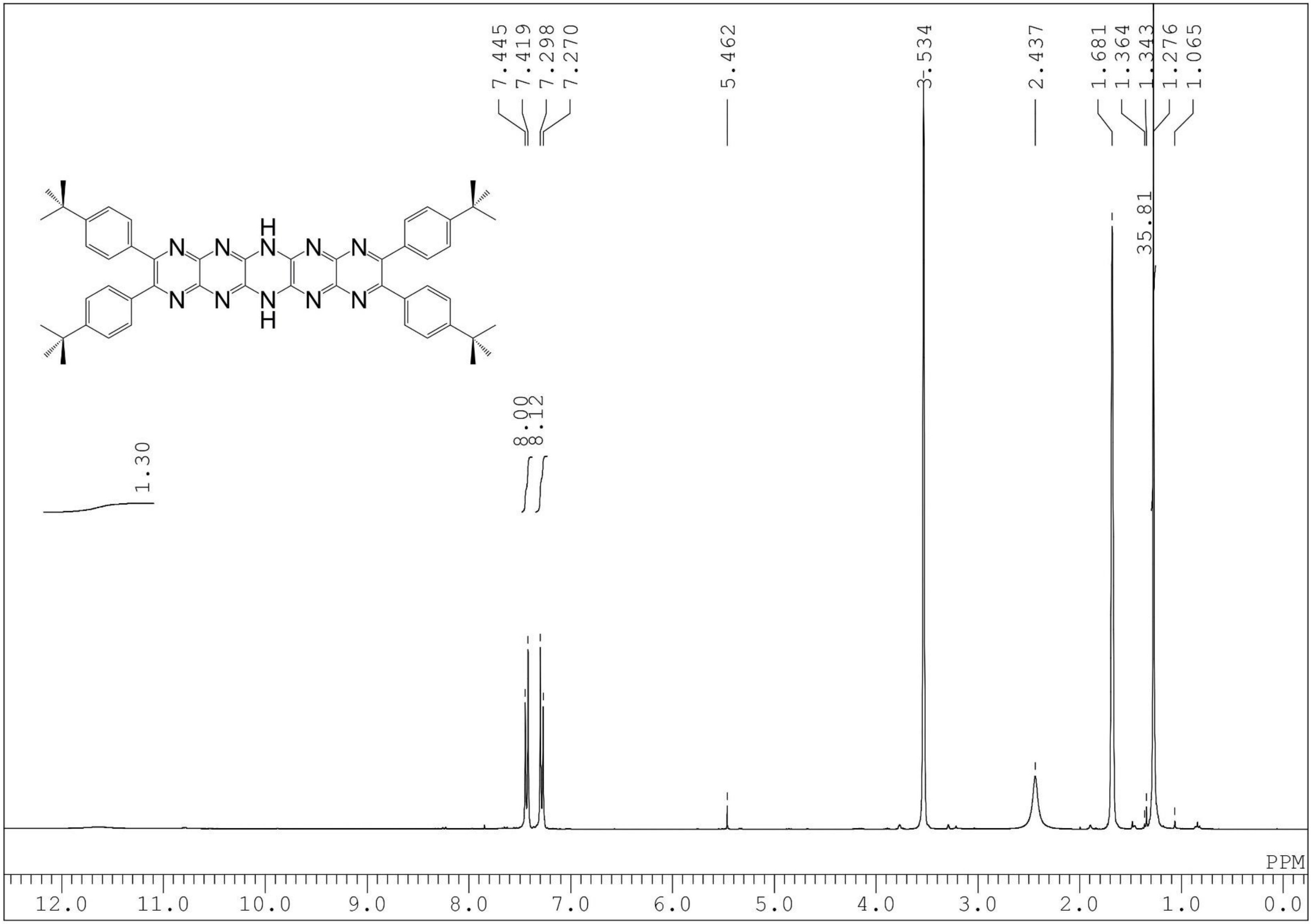

Supplementary Figure 41. ^{1}H-NMR spectrum of **1**-tBu in tetrahydrofuran-d_8.

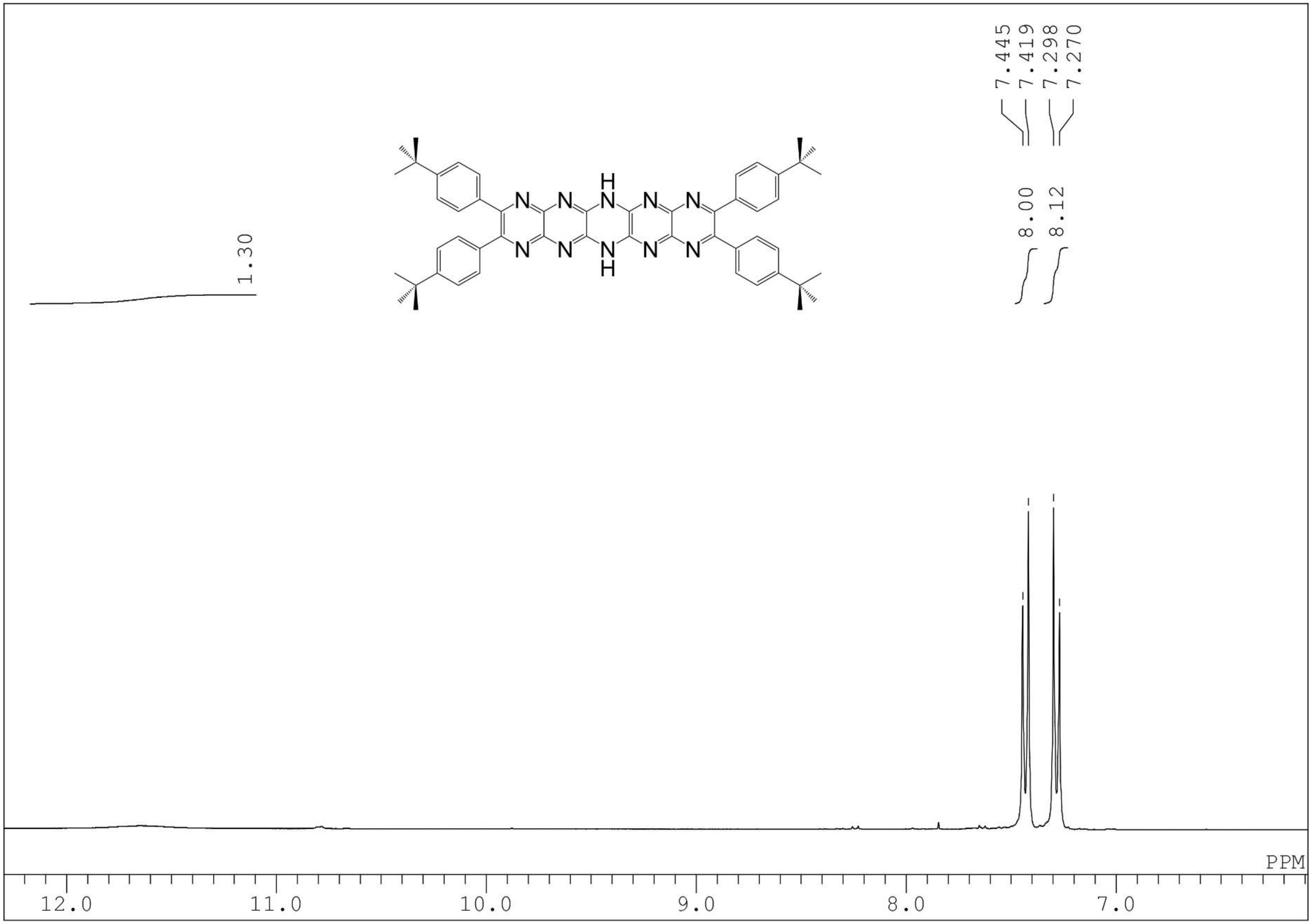

Supplementary Figure 42. Low field region of ^{1}H-NMR spectrum of **1**-tBu in tetrahydrofuran-d_8.

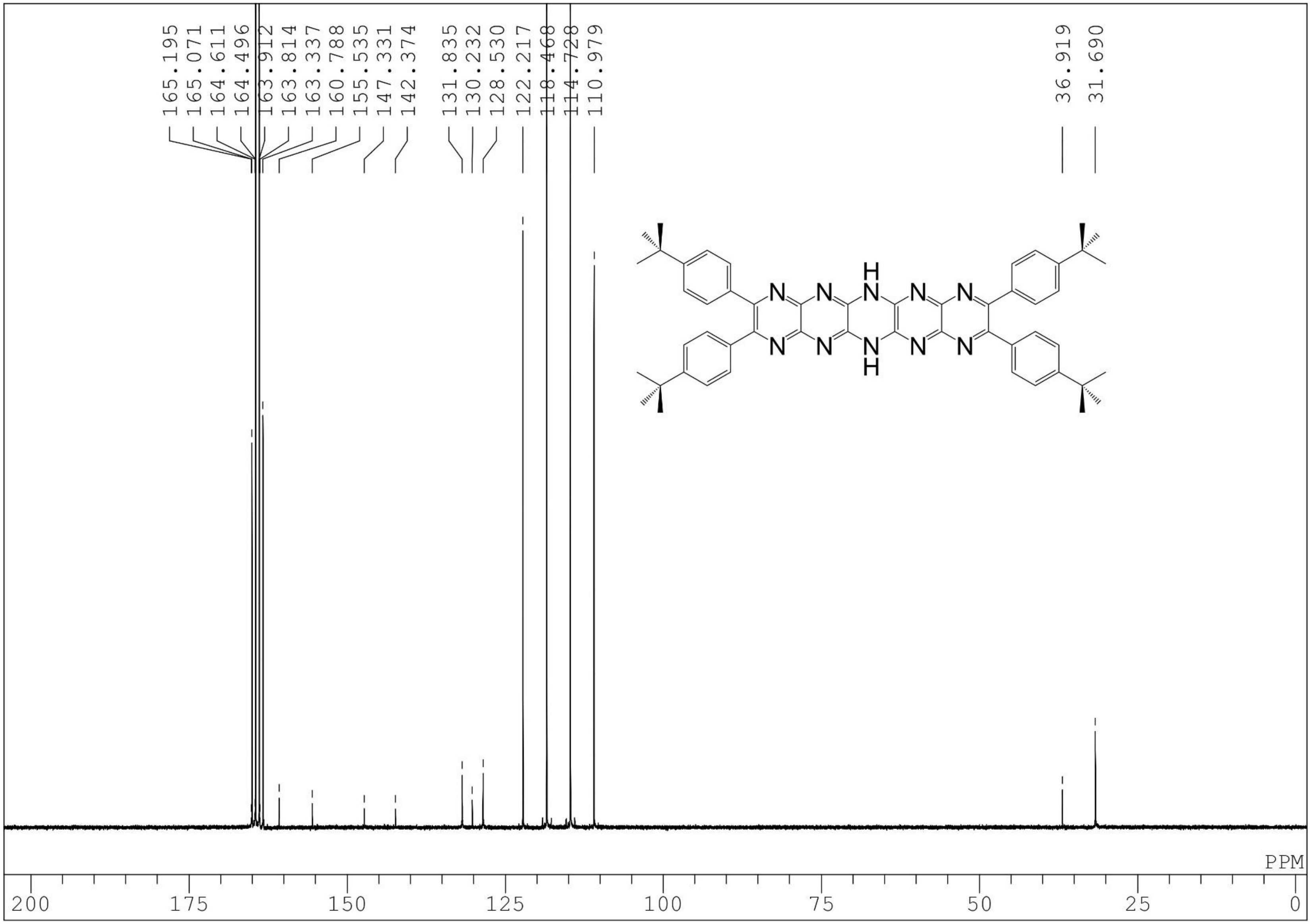

Supplementary Figure 43. ^{13}C-NMR spectrum of **1**-tBu in trifluoroacetic acid-d_1.

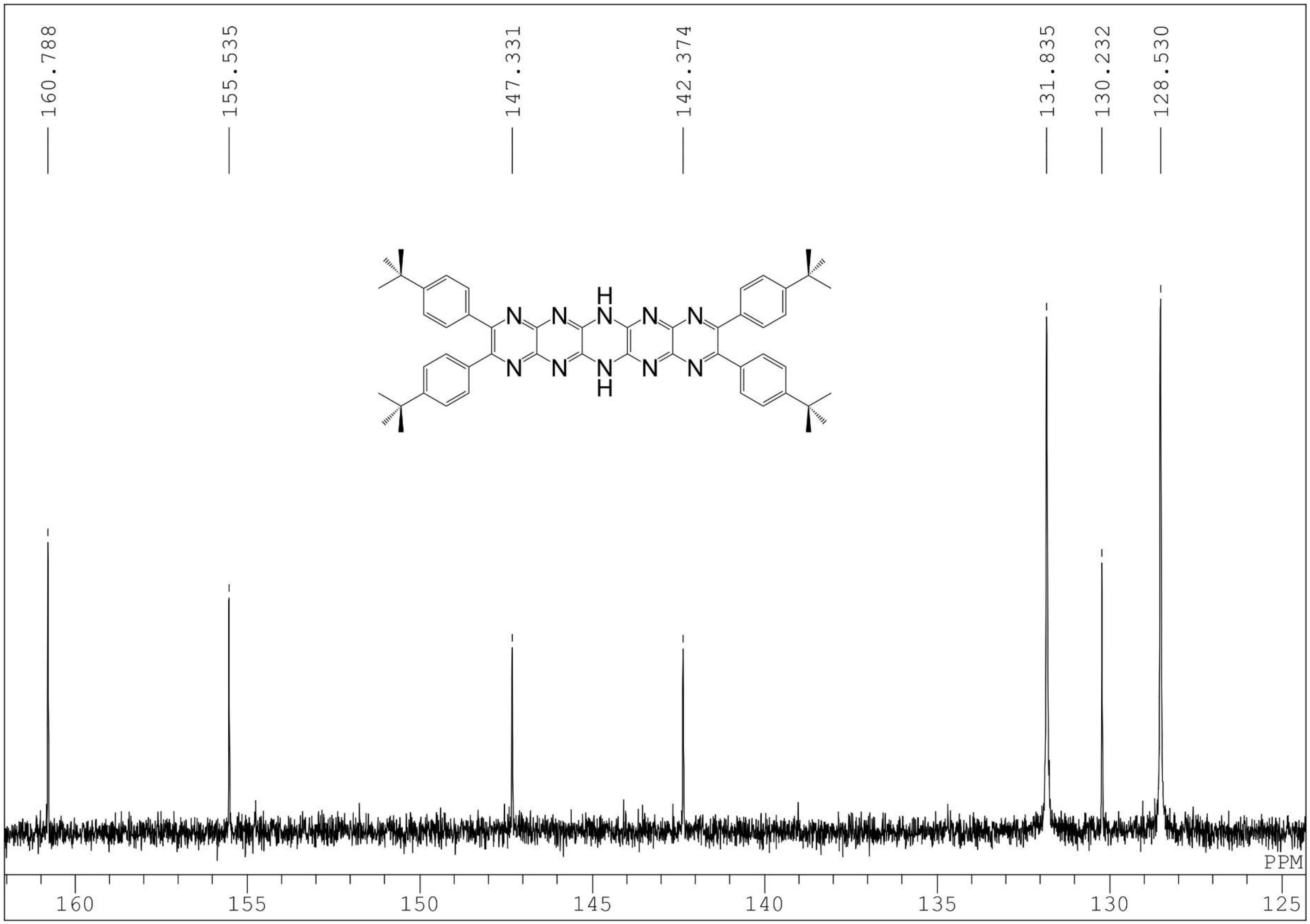

Supplementary Figure 44. Aromatic region of ^{13}C-NMR spectrum of **1**-tBu in trifluoroacetic acid-d_1.

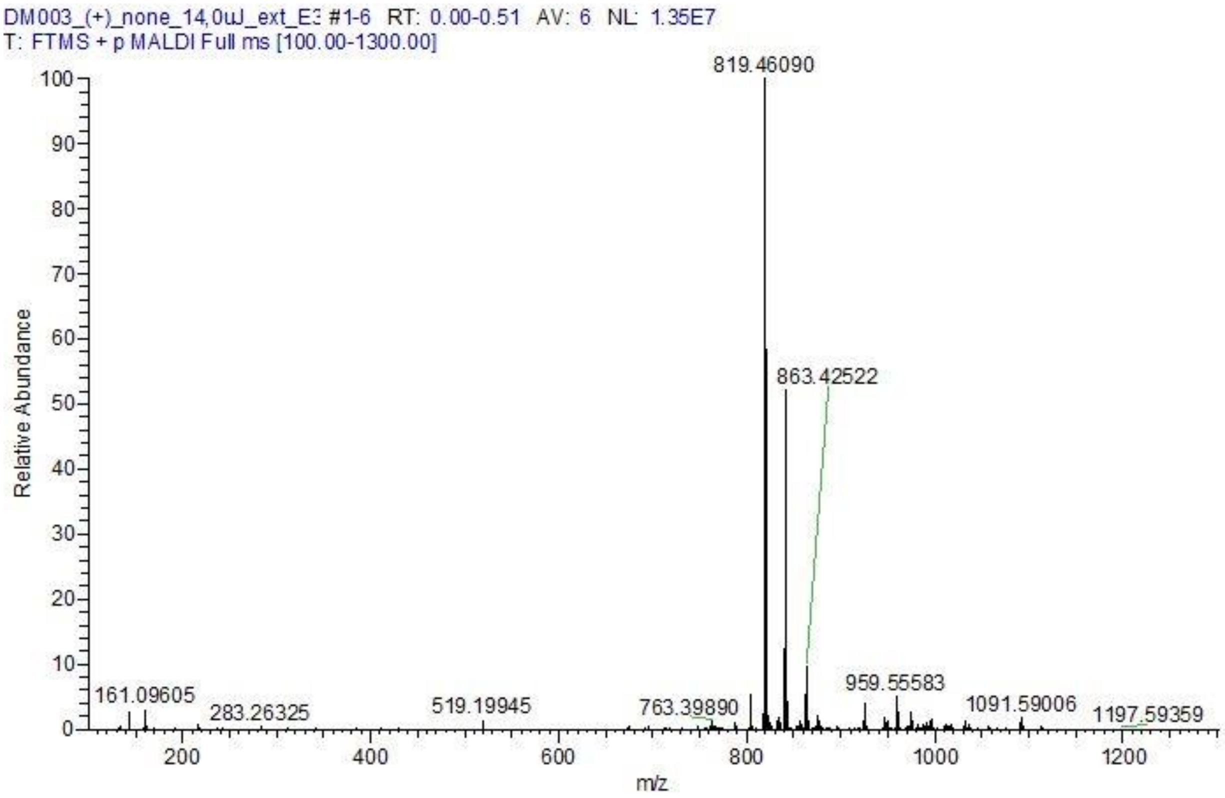

Supplementary Figure 45. High resolution MALDI-TOF mass spectrum of **1**-tBu.

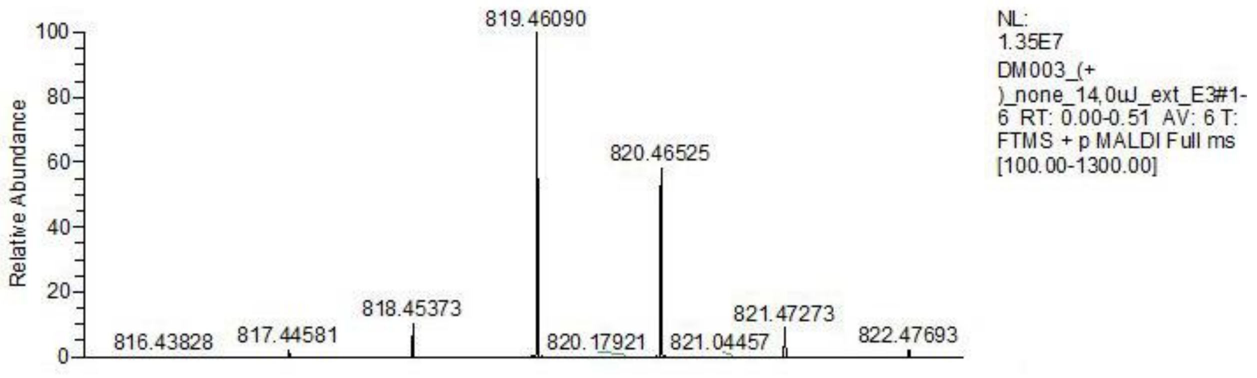

Supplementary Figure 46. Expansion of high resolution MALDI-TOF mass spectrum of **1**-tBu.

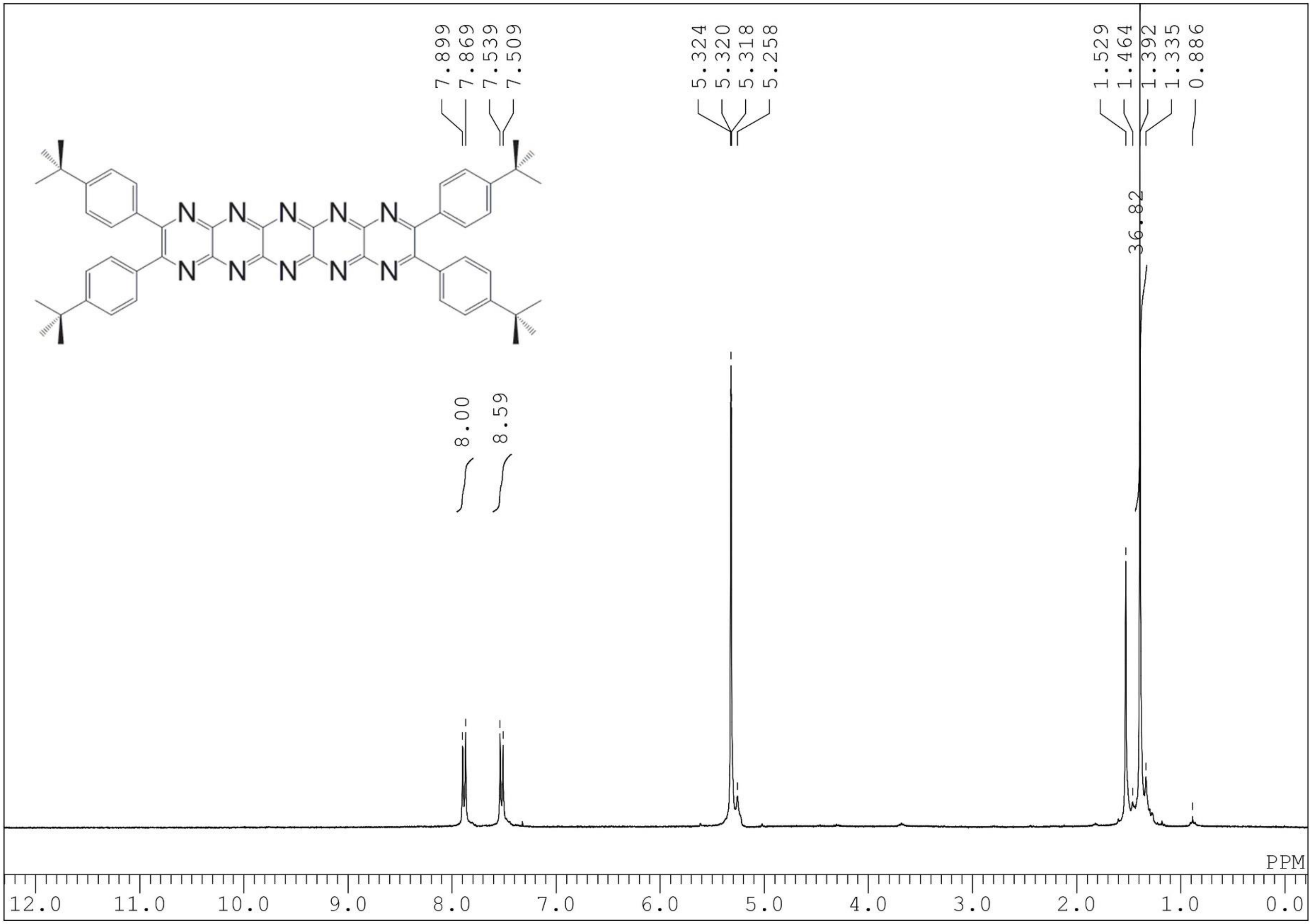

Supplementary Figure 47. ^{1}H-NMR spectrum of **1**-tBu-ox in CD_2Cl_2.

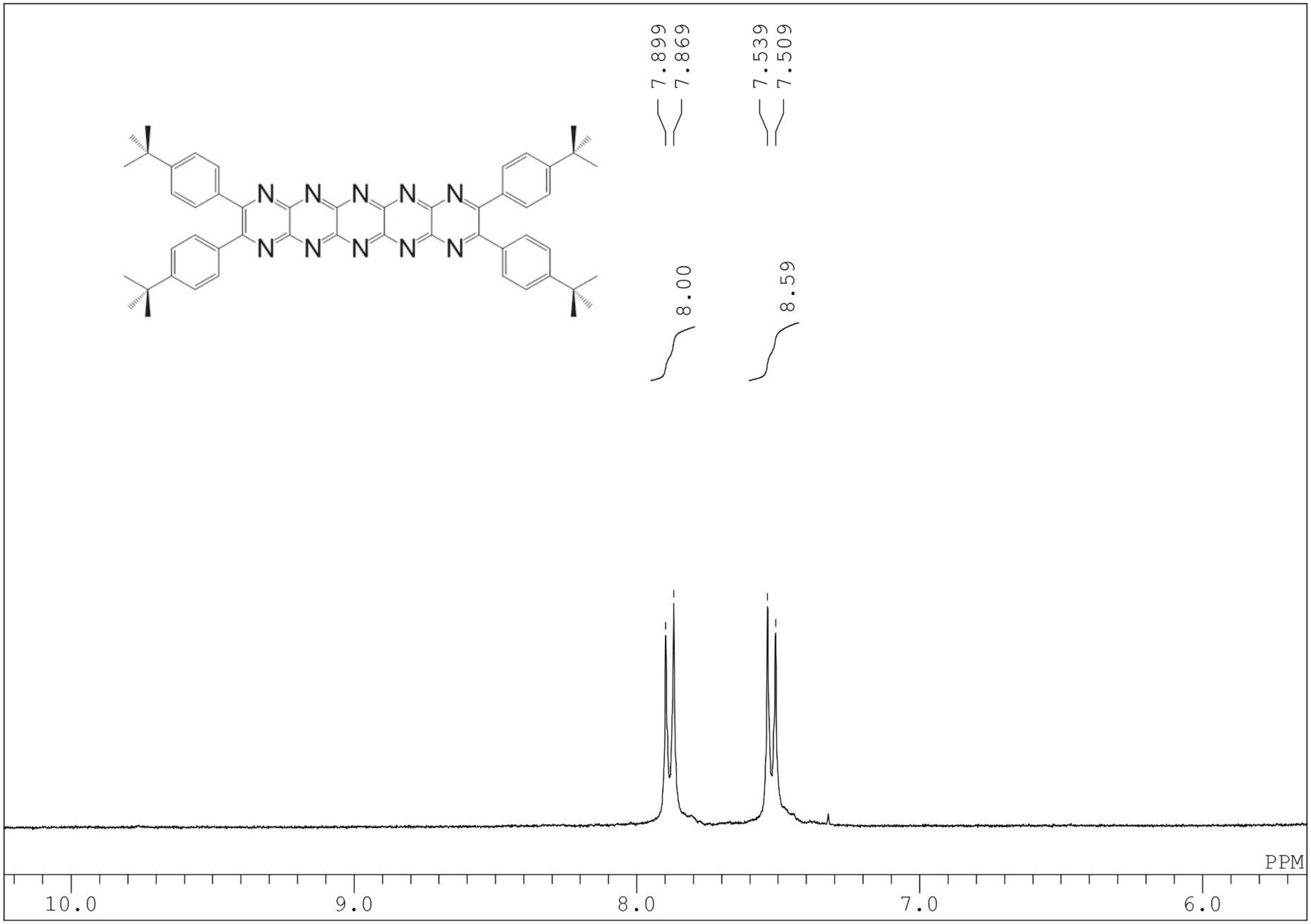

Supplementary Figure 48. Low field region of ^{1}H-NMR spectrum of **1**-tBu-ox in CD_2Cl_2.

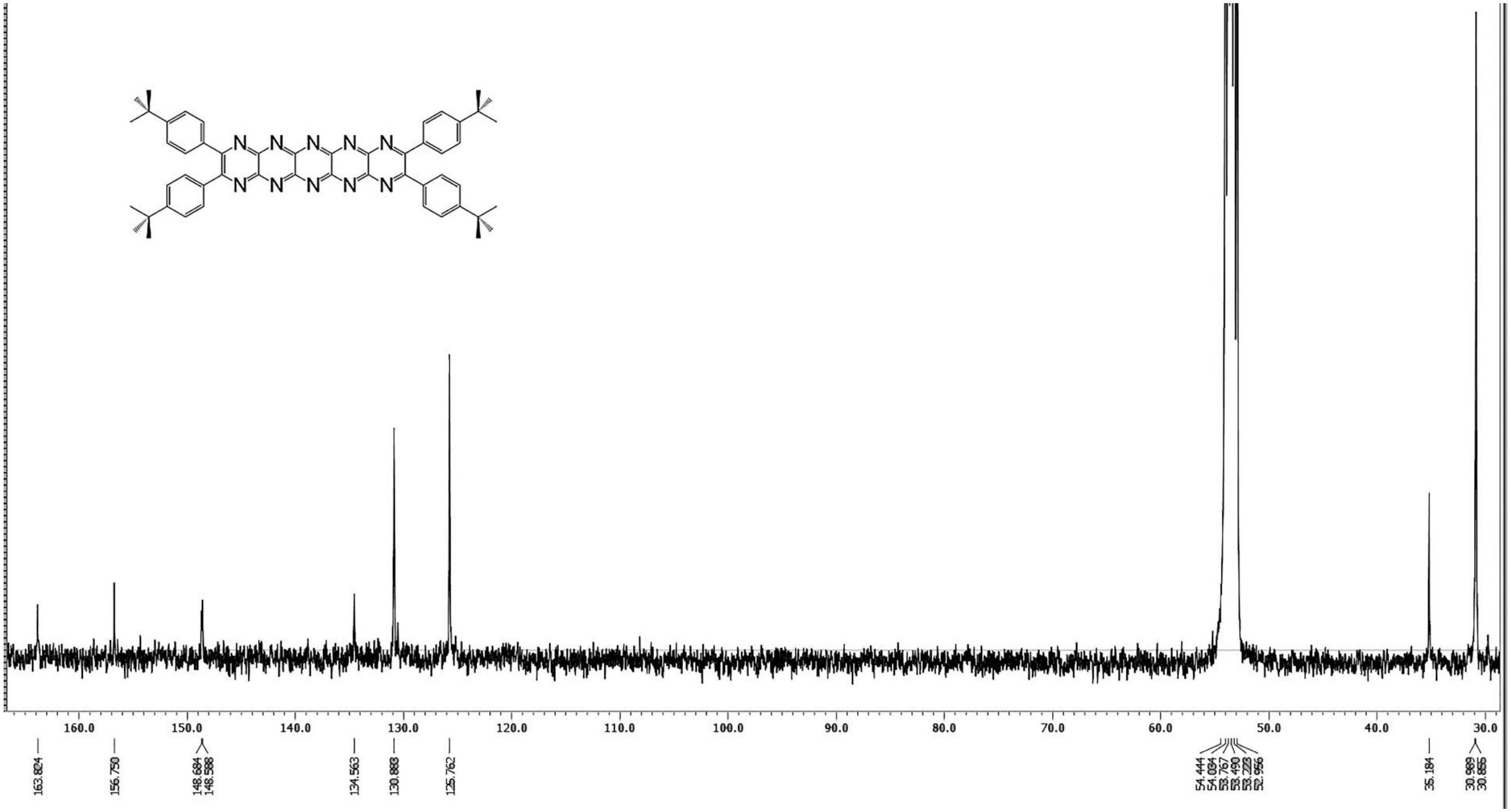

Supplementary Figure 49 ^{13}C-NMR spectrum of **1**-tBu-ox in CD_2Cl_2.

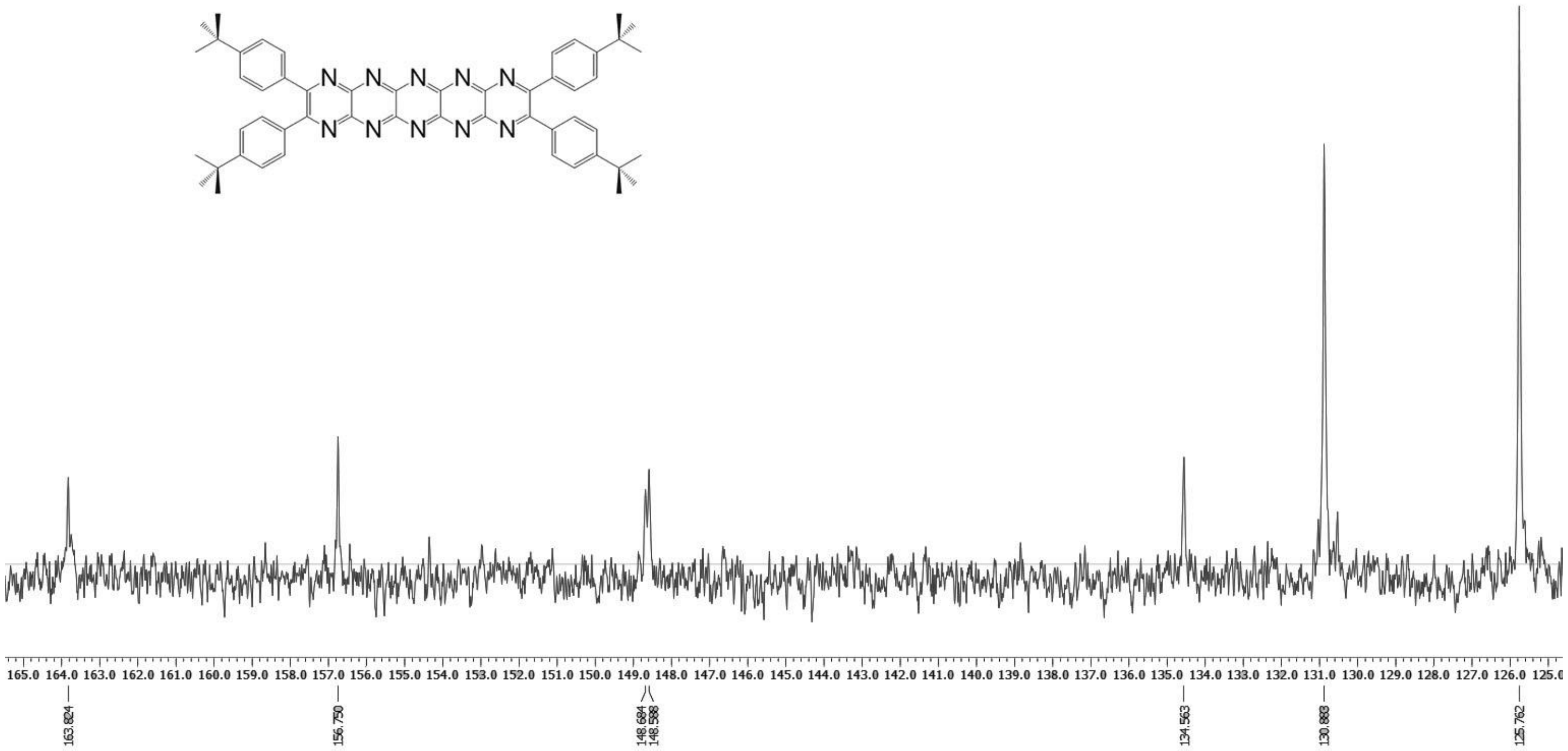

Supplementary Figure 50. Aromatic region of ^{13}C-NMR spectrum of **1**-tBu-ox in CD_2Cl_2.

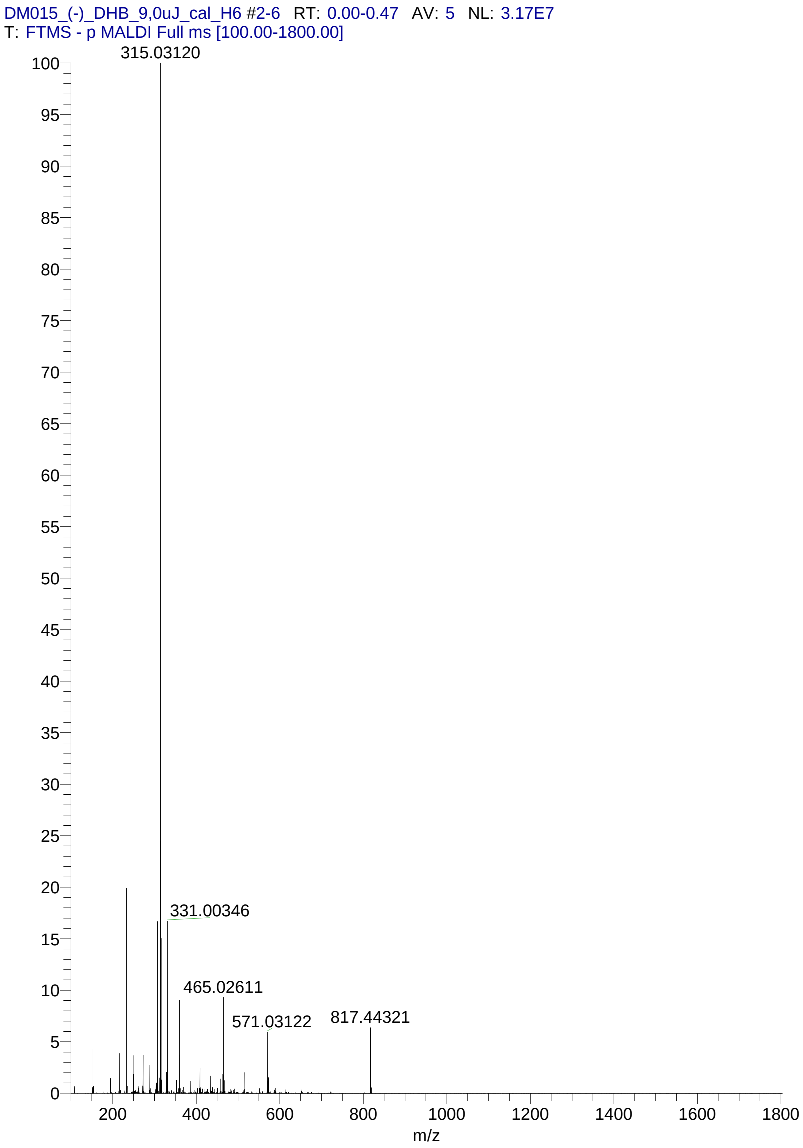

Supplementary Figure 51. High resolution MALDI-TOF mass spectrum of **1**-tBu-ox.

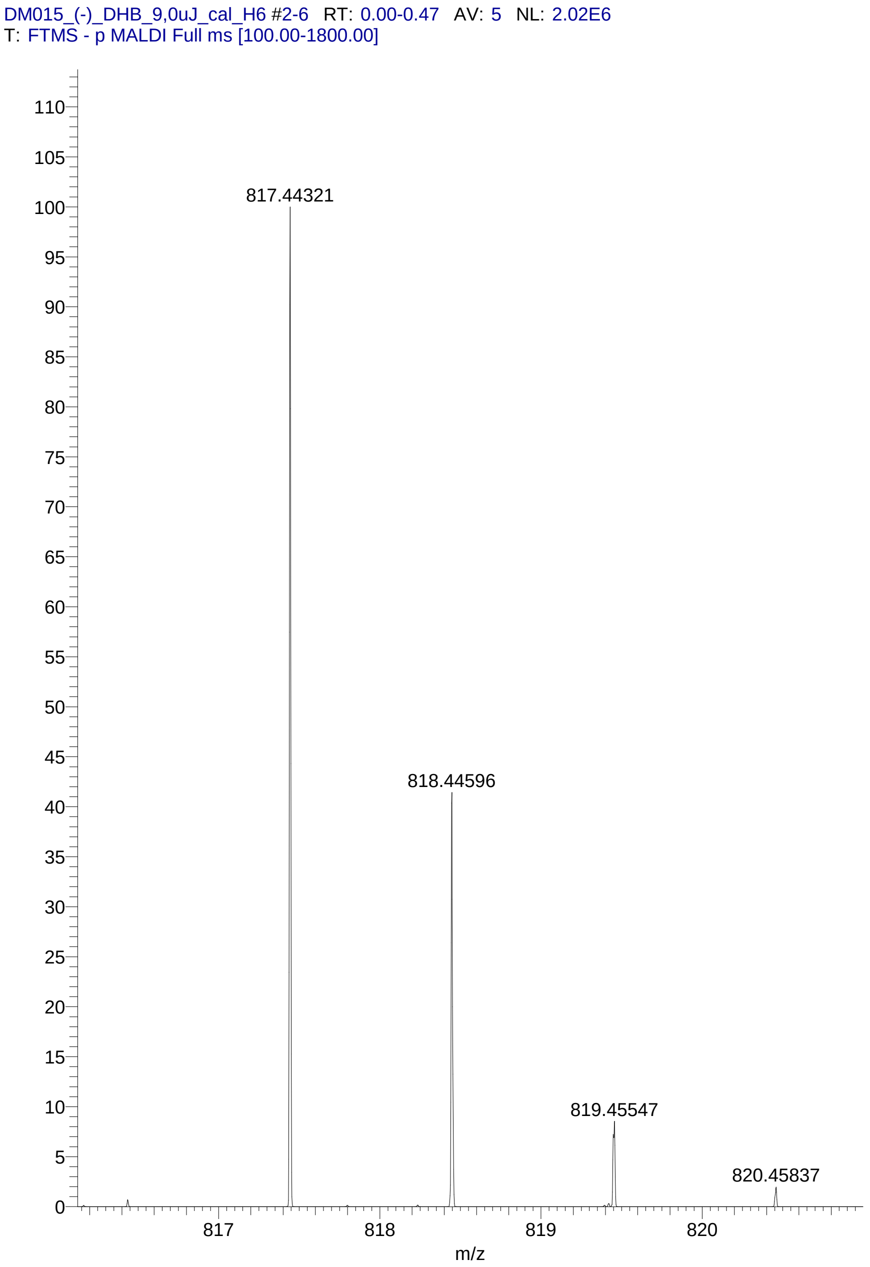

Supplementary Figure 52. Expansion of high resolution MALDI-TOF mass spectrum of **1**-tBu-ox.

Supplementary References

S1. Bickelhaupt, F. M., *et al.*, The carbon-lithium electron pair bond in $(CH_3Li)_n$ (n = 1, 2, 4). *Organometallics* **15**, 2923–2931 (1996). DOI: 10.1021/om950966x

S2. Fonseca Guerra, C., Handgraaf, J.-W., Baerends, E. J., Bickelhaupt, F. M. Voronoi deformation density (VDD) charges: assessment of the Mulliken, Bader, Hirschfeld, Weinhold, and VDD methods for charge analysis. *J. Comp. Chem*. 25, 189–210 (2003). DOI: 10.1002/jcc.10351

S3. Zhao, Y., *et al*. Dicyanopyrazine-derived push-pull chromophores for highly efficient photoredox catalysis. *RSC Adv.* **4**, 30062–30067 (2014). DOI: 10.1039/C4RA05525J

S4. Liu, X., Ye, X., Bureš, F., Liu, H. & Jiang, Z. Controllable chemoselectivity in visible-light photoredox catalysis: four diverse aerobic radical cascade reactions. *Angew. Chem. Int. Ed.* **54**, 11443–11447 (2015). DOI: 10.1002/anie.201505193.

S5. Zhang, C., *et al*. Visible light photocatalytic aerobic oxygenation of indoles and pH as a chemoselective switch. *ACS Catal.* **6**, 6853–6860 (2016). DOI**:** 10.1021/acscatal.6b01969

S6. Wei, G., *et al*. Enantioselective aerobic oxidative C(sp^3)-H olefination of amines via cooperative photoredox and asymmetric catalysis. *ACS Catal.* **6**, 3708–3712 (2016). DOI**:** 10.1021/acscatal.6b00846

S7. Hloušková, Z., *et al*, *ChemistrySelect,* **3**, 4262–4270 (2018). DOI: 10.1002/slct.201800719

S8. Liu, D., *et al*. Design, synthesis, and application of highly reducing organic visible-light photocatalysts. *Org. Lett.* **20**, 5700–5704 (2018). DOI**:** 10.1021/acs.orglett.8b02420

S9. Condie, A. G., Gonzáles-Gómez, J. C. & Stephenson C. R. J. Visible-light photoredox catalysis: aza-Henry reactions via C-H functionalization. *J. Am. Chem. Soc.* **132**, 1464–1465 (2010). DOI**:** 10.1021/ja909145y
