# Supplementary material for: Pyrazinacenes exhibit on-surface oxidation-state-dependent conformational and self-assembly behaviours
Source: Commun Chem. 2021 Mar 10;4:29. doi: 10.1038/s42004-021-00470-w (PMC9814942; doi:10.1038/s42004-021-00470-w)
Supplement: Supplementary file 2 — Description of Additional Supplementary Files [file 42004_2021_470_MOESM2_ESM.pdf]

Description of Additional Supplementary Files

File name: Supplementary Data 1

Description: Crystallographic information file (Cif) of compound 2.

File name: Supplementary Data 2

Description: Atomic coordinates of 1 (in vacuum).

File name: Supplementary Data 3

Description: Atomic coordinates of 1-ox (in vacuum).

File name: Supplementary Data 4

Description: Atomic coordinates of 1-ox2 (in vacuum).

File name: Supplementary Data 5

Description: Atomic coordinates of 2 (in vacuum).

File name: Supplementary Data 6

Description: - Atomic coordinates of 2-ox (in vacuum).

File name: Supplementary Data 7

Description: Atomic coordinates of 2-ox2 (in vacuum).

File name: Supplementary Data 8

Description: Atomic coordinates of 2 (on Cu(111)).

File name: Supplementary Data 9

Description: Atomic coordinates of 2-ox (on Cu(111)).

File name: Supplementary Data 10

Description: Atomic coordinates of 2-ox2 (on Cu(111)).

File name: Supplementary Data 11

Description: Atomic coordinates of 2-ox (two adjacent molecules on Cu(111)).
